# mRNP granule proteins Fmrp and Dcp1a differentially regulate mRNP complexes to contribute to control of muscle stem cell quiescence and activation

**DOI:** 10.1186/s13395-021-00270-9

**Published:** 2021-07-08

**Authors:** Nainita Roy, Swetha Sundar, Malini Pillai, Farah Patell-Socha, Sravya Ganesh, Ajoy Aloysius, Mohammed Rumman, Hardik Gala, Simon M. Hughes, Peter S. Zammit, Jyotsna Dhawan

**Affiliations:** 1 Institute for Stem Cell Science and Regenerative Medicine, Bangalore, India; 2 Centre for Cellular and Molecular Biology, Hyderabad, India; 3 National Center for Biological Sciences, Bangalore, India; 4 Manipal Academy of Higher Education, Manipal, India; 5 King’s College London, Randall Centre for Cell & Molecular Biophysics, New Hunt’s House, Guy’s Campus, London, UK

**Keywords:** Quiescence, mRNP granule, Translational control, mRNA decay, Skeletal muscle, Myoblast, G0, Fmrp, Dcp1a, *Fmr1* knockout, Muscle stem cell

## Abstract

**Background:**

During skeletal muscle regeneration, satellite stem cells use distinct pathways to repair damaged myofibers or to self-renew by returning to quiescence. Cellular/mitotic quiescence employs mechanisms that promote a poised or primed state, including altered RNA turnover and translational repression. Here, we investigate the role of mRNP granule proteins Fragile X Mental Retardation Protein (Fmrp) and Decapping protein 1a (Dcp1a) in muscle stem cell quiescence and differentiation.

**Methods:**

Using isolated single muscle fibers from adult mice, we established differential enrichment of mRNP granule proteins including Fmrp and Dcp1a in muscle stem cells vs. myofibers. We investigated muscle tissue homeostasis in adult Fmr1-/- mice, analyzing myofiber cross-sectional area in vivo and satellite cell proliferation ex vivo. We explored the molecular mechanisms of Dcp1a and Fmrp function in quiescence, proliferation and differentiation in a C2C12 culture model. Here, we used polysome profiling, imaging and RNA/protein expression analysis to establish the abundance and assembly status of mRNP granule proteins in different cellular states, and the phenotype of knockdown cells.

**Results:**

Quiescent muscle satellite cells are enriched for puncta containing the translational repressor Fmrp, but not the mRNA decay factor Dcp1a. MuSC isolated from *Fmr1*^*-/-*^ mice exhibit defective proliferation, and mature myofibers show reduced cross-sectional area, suggesting a role for Fmrp in muscle homeostasis. Expression and organization of Fmrp and Dcp1a varies during primary MuSC activation on myofibers, with Fmrp puncta prominent in quiescence, but Dcp1a puncta appearing during activation/proliferation. This reciprocal expression of Fmrp and Dcp1a puncta is recapitulated in a C2C12 culture model of quiescence and activation: consistent with its role as a translational repressor, Fmrp is enriched in non-translating mRNP complexes abundant in quiescent myoblasts; Dcp1a puncta are lost in quiescence, suggesting stabilized and repressed transcripts. The function of each protein differs during proliferation; whereas Fmrp knockdown led to decreased proliferation and lower cyclin expression, Dcp1a knockdown led to increased cell proliferation and higher cyclin expression. However, knockdown of either Fmrp or Dcp1a led to compromised differentiation. We also observed cross-regulation of decay versus storage mRNP granules; knockdown of Fmrp enhances accumulation of Dcp1a puncta, whereas knockdown of Dcp1a leads to increased Fmrp in puncta.

**Conclusions:**

Taken together, our results provide evidence that the balance of mRNA turnover versus utilization is specific for distinct cellular states.

**Supplementary Information:**

The online version contains supplementary material available at 10.1186/s13395-021-00270-9.

## Background

During skeletal muscle regeneration, the resident muscle stem cells, called satellite cells (MuSc), use distinct pathways to either enter myogenic differentiation to restore functional tissue, or self-renew by returning to mitotic quiescence to replenish the stem cell compartment. We have previously reported transcriptional and epigenetic mechanisms that control the choice between these irreversible and reversible cell cycle arrests [1–3]. In particular, quiescence is regulated by mechanisms that promote a poised or primed state, compatible with re-entry into the cell cycle [4]. The view of quiescence (G0) as an actively managed poised state, rather than an inert default state, is supported by several findings [5], which show that two major programs (the cell cycle and myogenesis) are held in abeyance by diverse mechanisms [6–8]. In addition to transcriptional and epigenetic silencing in G0, quiescent cells also exhibit translational repression [9], but remain capable of rapid remobilization of pre-existing transcripts onto polysomes during cell cycle activation.

Upon export from the nucleus, newly synthesized mRNAs are either rapidly assembled onto polysomes for immediate translation or held in a non-translating compartment, bound by a variety of RNA-binding proteins that control mRNA transport, localization, decay, and translational efficiency. RNA-binding proteins dynamically coalesce, along with mature mRNAs and miRNAs, into mRNP granules [10, 11]. Depending on the cellular context and lineage, several kinds of mRNP granules exist [10] that, due to their distinct composition, may function differently to regulate mRNA utilization [12]. Among mRNP granules, the best studied are P-bodies and stress granules. P-bodies are dynamic structures that are enriched in proteins involved in mRNA decay (such as Dcp1a, Edc4, Edc3, Lsm1-7 complex) [13, 14]. In contrast, stress granules may form in response to stress and contain stalled translation initiation complexes (containing Fmrp, eIF-4E, eIF-4G, Pabp, Tia-1/TiaR), and occasionally, 40S ribosomal subunits [15]. P-bodies and stress granules share many proteins and interact with each other, precluding unambiguous classification based [15] on the presence or absence of individual components, and prompting the use of the inclusive nomenclature of “mRNP granules”. Recent reports of mRNP granule proteins in MuSC suggest a role for translational control and use of stored mRNA in regulation of quiescence and activation [16, 17] and in regeneration [18].

The flux of transcripts between mRNP granules is associated with cell state transitions, and altered aggregation status is reported in neuromuscular diseases, the best-studied example being fragile X syndrome (FXS). In this devastating neuro-developmental disorder, loss of the fragile X mental retardation protein (Fmrp) leads to a spectrum of autistic features characterized by cognitive and behavioral deficits [19]. The location of Fmrp in cytoplasmic granules and its molecular function as a repressor of activity-dependent protein translation in axons [20] suggest mechanisms by which signal-dependent protein synthesis is required for higher-level brain function [21]. Despite the nearly ubiquitous expression of Fmrp [17], little is known of its specific role in non-neuronal tissues, including development and regeneration of skeletal muscle. Fmrp has been reported to be downregulated during muscle differentiation [22] and is detected in quiescent MuSC, where it regulates MuSC function via control of the myogenic determinant *Myf5* mRNA [17, 23].

Increasing evidence points to a role for post-transcriptional control in quiescent cells, including quiescent adult MuSC. Studies in yeast and cultured fibroblasts showed that Fmrp and the related Fxr1 are important for entry into G0 [24]. In myoblasts, the entry into mitotic quiescence is associated with an induction of genes encoding mRNP granule components, such as tristetraprolin (TTP) [25, 26], primarily involved in AU-rich element (ARE)-mediated decay, that are also required for MuSC regenerative function [27]. A pioneering report by Crist *et.al.,* [17] showed that quiescent MuSC sequester transcripts of *Myf5* in an untranslated fraction, and remobilize them onto polysomes during reactivation. Further, a general repression of protein synthesis by phosphorylation of the translation initiation factor eIF2α is essential for maintenance of the quiescent state [9]. However, there is little information available on the composition and function of heterogeneous mRNP granules that might regulate the quiescent state per se, and in particular, the relative roles of translational repression and mRNA decay in the entry into quiescence. This balance may be important in the context of the global suppression of macromolecular synthesis, in G0 cells [16]. For example, key regulators of protein synthesis such as mTOR control awakening of quiescent MuSC [28], but the coupling of mRNA utilization to metabolic activation has not been extensively explored in MuSC.

MuSC function is intimately linked to the ability to enter and exit quiescence. Whereas both differentiation and quiescence are mitotically inactive states, muscle terminal differentiation is irreversible and requires preferential transcription and translation of tissue-specific proteins that comprise and control the specialized sarcomeric cytoskeleton [16]. By contrast, quiescence is reversible and is characterized by a broad suppression of the differentiation program and increased expression of the MuSC-specific transcription factor Pax7, which are reversed during cell cycle activation, along with re-induction of determination factors MyoD and Myf5 [26]. Indeed, quiescent MuSC exhibit translational control of lineage determinants [27], with *Myf5* transcripts held in non-translating mRNPs [17, 23].

Prompted by our observation that Fmrp mRNA expression is induced in G0 [1], in the present study we profiled expression of a set of mRNP proteins in muscle cells and explored their function in quiescence. We first surveyed the expression and distribution of mRNP complex proteins in MuSC versus myofibers in isolated single muscle fiber preparations *ex vivo*. We report the enrichment of Fmrp bodies in MuSC in wild-type mice and reveal a role for Fmrp in MuSC function *in vivo* using *Fmr1*^-/-^ mice, suggesting its involvement in homeostatic and regenerative control in muscle, beyond its established role in neuronal function. During primary MuSC activation on myofibers, we found that Fmrp and Dcp1a show reciprocal expression and organization, with Fmrp puncta prominent in quiescence, but Dcp1a puncta appearing during activation/proliferation. We also explored the muscle cell-intrinsic functions of Fmrp as distinct from neurological effects manifested *in vivo*, using a cultured myoblast model of quiescence. Our results suggest the existence of distinct mRNP complexes in different cellular states (proliferation, quiescence, and differentiation). Specifically, whereas translational repressive complexes containing Fmrp predominate in G0, we report an enrichment of nonsense-mediated mRNA decay complexes containing the mRNA-decapping enzyme 1A (Dcp1a) in proliferating myoblasts, suggesting that post-transcriptional regulatory complexes may be remodeled depending on cellular context. Functional analysis using mRNA knockdown indicates that Fmrp and Dcp1a play opposing roles in myogenic proliferation and quiescence; Fmrp sustains proliferative potential, whereas Dcp1a functions to restrain proliferation of myoblasts. Intriguingly, these opposing functions cross-regulate, such that knockdown of Fmrp leads to increased *Dcp1a* expression and assembly into puncta, and reciprocally, Dcp1a knockdown myoblasts show increased *Fmrp* expression and assembly into puncta. However, unlike their opposing roles in proliferation, knockdown of either Dcp1a or Fmrp led to compromised myogenic differentiation. Taken together, our study shows the importance of the balance between translational repression and mRNA decay in the regulation of quiescence, and indicates a role for distinct mRNP granule proteins in regulating this equilibrium.

## Materials and methods

### Single myofiber isolation and analysis

Animal experiments were carried out in accordance with CPCSEA guidelines of the Govt. of India as approved by the Institutional Animal Ethics Committee of InStem and CCMB, or in accordance with British law under provisions of the Animals (Scientific Procedures) Act 1986, as approved by the Ethical Review Process Committee of King’s College London.

**Fig. 1:**
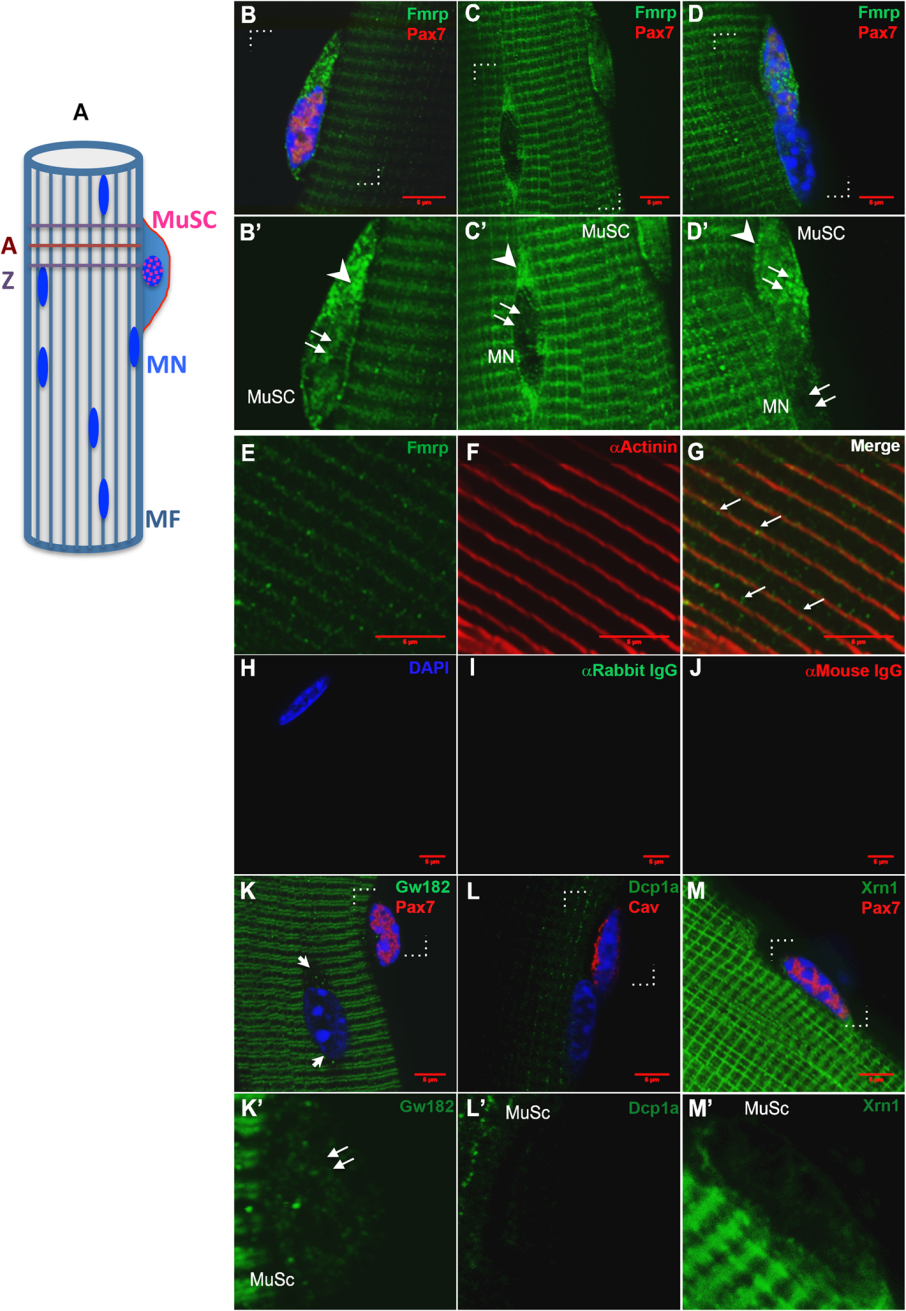
Expression and distribution of mRNP granule proteins in isolated skeletal muscle fibers**. A** Schematic of an isolated myofiber (MF) depicting myonuclei (MN) and an associated muscle satellite cell (MuSC). The longitudinal striations represent orientation of the myofibrils while the cross-striations represent the A-band (A) and Z line (Z). **B**–**D**, **B’**–**D’** Depict magnified views of the regions enclosed by brackets (dotted lines) in **B**–**D** to visualize subcellular distribution of Fmrp. Arrow heads indicate cytoplasm, double arrows indicate nucleus. Fmrp puncta are observed both in nucleus and cytoplasm of MuSC (**B**, **B’**) and as cross-striated staining in myofiber. Puncta also accumulate in a cytoplasmic domain adjacent to the MN, while MN is itself not stained (**C**, **C’**). Nuclear accumulation of Fmrp is also seen in the Pax7^**+**^ MuSC nucleus (**D**, **D’**) but not in an adjacent Pax7^**-**^ MN. **B’**, **C’**, and **D’** represent single-channel (488) images. **E**–**G** Distribution of Fmrp puncta (green) in myofiber in a cross-striated pattern congruent with Z lines revealed by α-actinin (red). Arrows in G point to Fmrp puncta co-localizing with α-actinin striations. **H–I**. Secondary antibody controls (mouse and rabbit) do not show either punctate or striated background. **K** Distinct GW182 bodies are visible in Pax7^**+**^ MuSC. Pax7^**-**^ MN also show distinct perinuclear puncta (arrowheads) and significant punctate staining is observed in MF cytoplasm in a doublet striated pattern likely reflecting A-band localization. **K’** Region within brackets in **k** magnified to show GW182 puncta in the MuSC nucleus (double arrow). **L**, **L’** No enrichment (either nuclear or cytoplasmic) is detected of Dcp1a in MuSC nucleus (marked with the membrane marker Caveolin 1). Faint fibrillar puncta are observed in myofibers. **M, M’** Xrn1 is faintly detected in MuSC, but strongly expressed in myofibres in both a longitudinal and cross-striated pattern. **K’**, **L’**, and **M’** represent single channel (green) images of enlarged areas indicated by brackets in **K**, **L**, and **M**, respectively

**Fig. 2:**
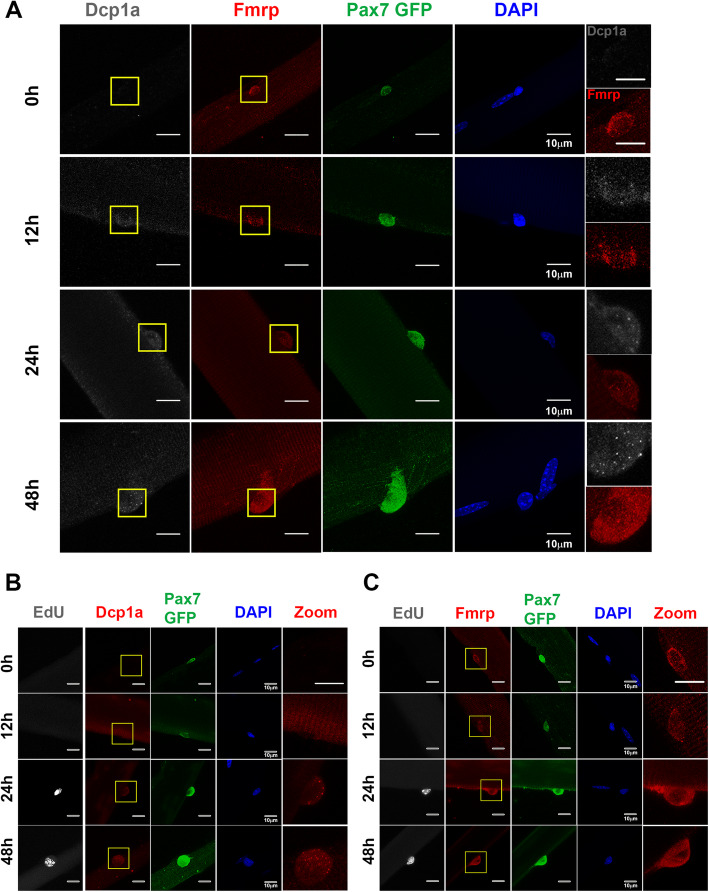
Dynamics of Fmrp and Dcp1a granules in quiescent and activated MuSCs on isolated single muscle fibers**. a**–**c** Immunofluorescence analysis of Dcp1a and Fmrp in freshly isolated EDL myofibers (0 h) and at 12, 24, and 48 h of culture. MuSCs are marked with Pax7-nGFP. **a** Fmrp puncta are evident in quiescent MuSCs at 0 h of culture, and become dispersed at 24 h, whereas Dcp1a puncta are not evident until 24 h of activation. The patterns of staining are consistent with reciprocal patterns of Dcp1a and Fmrp assembly into granules. **b** The pattern of Dcp1a in proliferating MuSCs (Pax7-nGFP^**+**^EdU^**+**^) confirms the timing of MuSC activation. Dcp1a puncta are not observed in quiescent Pax7^**+**^EdU^**-**^ cells, but are present in activated Pax7^**+**^EdU^**+**^ MuSC at 24 and 48 h. **c** By contrast to Dcp1a, the pattern of Fmrp is punctate in quiescent MuSC (Pax7^+^EdU^-^) and becomes diffuse in activated Pax7^+^EdU^+^ MuSC at 24 and 48 h. Immunofluorescence was performed in *N* = 3 biological replicates using > 5 EDL fibers for each combination of antibodies in each assay. Scale bars are 10 μm, or 4.8 μm in magnified panels.

EDL muscles were dissected from hind limbs of 2–7-month-old mice of either sex (two C57BL/6 mice aged 8–10 weeks (Fig. 1) or six Pax7-nGFP mice (four males aged 3 months) and two females aged 5 months (Fig. 2) [29]. Isolated muscles were digested with 400 U/ml Type I collagenase (Worthington) in DMEM at 37 °C, till single fibers dissociated. All dissociated fibers were transferred into fresh DMEM medium and triturated gently to release individual fibers using fire-polished pasteur pipettes. Dispersed single fibers were either immediately fixed in in 4% paraformaldehyde (PFA) for 10 min or cultured for up to 48 h in DMEM, 10% Horse Serum, 20% Fetal Bovine Serum, and 2% Chick Embryo Extract (Sera Lab, CE-650-J) followed by fixation, washed three times with PBS, picked and placed on charged slides (Thermo-Fisher) for immunostaining. Fibers were permeabilized with 0.5% Tween-20 in PBS for 1 h, blocked in 5% BSA in PBS 0.5% Tween-20 for 1 h. Subsequent steps were as for cultured cells. Samples were imaged on a LSM510 Meta or LSM 880 Airy Scan (Zeiss). Image analysis was done using ImageJ.

### Muscle histology

To determine muscle cross-sectional area, 2 male adult mice (6–8 weeks) each for *Fmr1*^-/-^ [30] and age-matched WT mice were used. The TA muscle was carefully dissected intact, fixed for 2 h in 4% PFA at 4 °C and equilibrated overnight in 20% sucrose at 4 °C. The muscle tissues were mounted in OCT in cryomoulds and flash frozen in a liquid nitrogen-cooled isopentane bath. Serial 20 μm cryosections were collected and immunolabelled with anti-laminin antibody to highlight the individual fiber perimeters, and imaged by confocal microscopy. For calculating the cross-sectional area (CSA) of myofibers, 7 sections were chosen at random from wild-type muscle and a corresponding section selected from *Fmr1* ^-/-^ muscle (from the equivalent position in the TA). The CSA of ~ 250 myofibers was measured from confocal images (LSM510 Meta) of sections using ImageJ software, and the mean CSA and two-tailed paired Student’s t test were performed to compare the difference between the two groups.

### Isolation of mouse muscle satellite cells

Primary MuSCs were purified from adult mice as described [31]. Briefly, all hind limb muscle groups were dissected from 6-week-old WT or *Fmr1*^-/-^ mice (1 male mouse per genotype), minced, and digested in collagenase type II (Cat# LS4196 Worthington Biochemical, 400 U/ml final concentration) for 90 min at 37 °C with gentle vortexing after every 15 min. The digested muscle slurry was filtered through 40-μm nylon mesh. The single cell suspension was treated with 0.8% ammonium chloride to lyse RBCs. Muscle mononuclear cells were washed twice with PBS and stained with biotinylated anti-VCAM-1 (BD Biosciences, Cat#553331) primary antibody for 30 min, washed with PBS and stained with Streptavidin, Alexa Fluor-488 conjugate (Invitrogen, Cat#S-11223) and CD45-PE (BD Biosciences, Cat#553081) conjugated antibody. Cell sorting was performed on Moflo XPD cytometer using gates for the VCAM-1^+^ and CD45^-^ population. The gated cell population was sorted directly into growth medium for subsequent culturing on Matrigel (BD Biosciences, Cat#354230) coated dishes for 6 days.

### Cell culture

The C2C12 mouse muscle cell line was cultured in DMEM supplemented with 20% FBS (with Penicillin/Streptomycin). To generate synchrony in G0 (reversible arrest), cells were cultured in methylcellulose suspension with 20% FBS for 48 h [32–34]. To differentiate cells into irreversibly arrested multinucleated myotubes, myoblasts at 80% confluency were cultured in low serum media (DMEM + 2% Horse serum) for 120 h with daily medium changes; myotubes appeared by day 2, with fusion increasing till day 5.

### Western blot analysis

Cells were lysed in 50 mM Tris HCl, pH 8, 150 mM NaCl, 5 mM MgCl_2_, 0.1% Nonidet P-40) supplemented with complete protease inhibitor cocktail (Roche Diagnostics, France) and incubated on ice for 30 min. Soluble proteins were recovered after centrifugation at 15,000 *g* at 4 °C for 10 min and quantified by the BCA method. Proteins were separated on a 4–12% polyacrylamide SDS-PAGE gel along with a pre-stained protein ladder (12–250 kDa) and transferred to a nitrocellulose membrane. Non-specific protein binding sites were blocked by incubation in 5% (w/v) non-fat dry milk (made in TBST), for 1 h at room temperature. The membrane was then incubated with antibodies against the different mRNP granule proteins overnight at 4 °C. After washing in TBST, the blot was incubated with horseradish peroxidase-conjugated goat anti-mouse or anti-rabbit IgG—for 45 min at room temperature. After washing in TBST, the blots were developed using chemiluminescence solutions and imaged using Image Quant (Antibody information in Table 3).

### Immunofluorescence and confocal analysis

Cells were grown on cover slips placed in 6-well plates (5 × 10^3^ cells per 18-mm cover slip). The next day, cells were rinsed with ice-cold PBS and fixed with 4% PFA for 10 min at room temperature followed by permeabilization with 0.1% Triton X-100. The cells were subjected to immunofluorescence staining with different antibodies overnight at 4 °C. The cells were washed with cold PBS and incubated with anti-Rabbit Alexa 488 (Invitrogen A11034, 1:500) and anti-Mouse Alexa 568 (Invitrogen A11037, 1:500) secondary antibodies at room temperature for 1 h. The cells were examined by confocal microscopy using LSM510 (Zeiss, Germany). Co-localization was quantified using ImageJ software, after maximizing the intensity from all the Z-stacks for a particular image, and analyzed by selecting the puncta as ROI. Image intensity was calculated using Fiji (ImageJ) software and corrected mean intensity (CMI = Total intensity of signal − Area of signal × Mean background signal) was calculated for more than 75 cells. All data points were plotted in Box and whisker plot, and *p* value was calculated by two-tailed paired Student’s *t* test.

### RNA interference using siRNA

The following small interfering RNAs (siRNAs) from Dharmacon, Thermo Scientific were used for the study: siGENOME SMARTpool siRNAs against mouse Fmrp (M-045448-01-0005), mouse Dcp1a (M-065144-01-0005) and non-targeting siRNA pool #1 (D-001206-13-20); each pool represents 4 distinct siRNAs targeting different sequences in the same transcript. C2C12 myoblasts maintained in growth medium (DMEM + 20% Fetal bovine serum) were transfected with the siRNAs listed above using the Lipofectamine ® RNAiMAX Reagent (Invitrogen) according to the manufacturer’s instructions. Eighteen hours post-transfection, the cells were either induced to differentiate in low mitogen medium (DMEM + 2% Horse serum), for 2 days to form myotubes (MT) or were synchronized to G0 in suspension cultures (1.3% methylcellulose in growth medium) [33]. siRNA-transfected cultures were harvested 48 h after induction of myogenesis or quiescence and subjected to different analyses including EdU proliferation assay, western blotting for different cell cycle proteins (such as Cyclins A, B, D1, and E), p27, p21, and qRT-PCR analysis. Knockdown in these cells was confirmed by western blotting using anti-Fmrp and anti-Dcp1a antibodies (Table 3).

siRNA target sequences (smart pool of 4 siRNAs per transcript):

Fmrp: GAUUAUCACCUGAACUAUU,

GAUCUGAUGGGUUUAGCUA,

CGUCACUGCUAUUGAUUUA,

GAUCAUUCCCGAACAGAUA.

Dcp1a: CAACAGCUAUGGGUCUAGA,

GACAGUAGAAGAGUUAUUU,

GUAUAGAAAUGCAAGUUUG,

GAAGGGACGUUAUUUGUAU.

Non-targeting siRNA pool#1:

UAGCGACUAAACACAUCAA,

UAAGGCUAUGAAGAGAUAC,

AUGUAUUGGCCUGUAUUAG,

AUGAACGUGAAUUGCUCAA.

*Quantitative real-time RT-PCR* was performed on an ABI 7900HT thermal cycler (Applied Biosystems) using the SDS 2.1® ABI Prism software. cDNA was prepared from 1 μg total RNA using superscript II (Invitrogen) and used in SYBR-Green assay (Applied Biosystems). Each sample was isolated from three independent biological samples and analyzed in triplicate reactions. Amplicons were verified by dissociation curves and sequencing. Primer sequences are listed in the Supplementary Information. Relative abundance of different mRNAs in Fmrp and Dcp1a knockdown cells was calculated with reference to cells transfected with non-targeting siRNA and normalized to GAPDH levels. Fold change was calculated using differences in normalized cycle threshold value 2^−ΔΔct^.

Primer sequences used in this study:

Gapdh - F: 5’-ATCAACCGGGAAGCCCATCAC -3’

R: 5’- CCTTTTGGCTCCACCCTTCA- 3’

Cyclin D1- F: 5’-AAGTGCGTGCAGAAGGAGATTGTG-3’

R:5’ TCGGGCCGGATAGAGTTGTCAGT-3’

Cyclin A2- F: 5’-TTCTGGAAGCTGACCCATTC-3’

R: 5’-GGCAAGGCACAATCTCATTT-3’

Cyclin B1- F: 5’-ATGGACACCAACTCTGCAGCAC-3’

R: 5’-CTGTGCCAGCGTGCTGATCT-3’

Cyclin E1- F: 5’-TGTCCTCGCTGCTTCTGCTTTGTATCAT-3’

R: 5’-GGCTTTCTTTGCTTG GGCTTTGTCC-3’

Dcp1a -F:5’- CCAGCTGAAGCTCCTACCAC-3’

R:5’- CTGTGGGGTCAACCTGAGTT-3’

Fmr1 - F: 5’-AGGCTTGGCAGGGTATGGTA -3’

R:5’-TGTACGATTTGGTGGTGGTCT-3’

Fmr1 -F: 5’-AGAGGAGGAGGCTTCAAAGG-3’

R: 5’- AGAGGAGGAGGCTTCAAAGG-3’

Myogenin- F: 5’-TGGGCATGTAAGGTGTGTAAGA-3’

R: 5’-ACTTTAGGCAGCCGCTGGT-3’

Pax7 - F: 5’-CATGGTGGGCCATTTCCACT-3’

R: 5’-GGCCCGGGGCAGAACTAC-3’

p27 - F: 5’-TGCAGTCGCAGAACTTCGAA-3’

R: 5’-ACACTCTCACGTTTGACATCTTCCT-3’

p16 - F: 5’-CGAACTCGAGGAGAGCCATC-3’

R: 5’- CGTGAACGTTGCCCATCATC-3’

Myf5 - F: 5’-CCCCACCTCCAACTGCTCTG-3’

R: 5’-CCAAGCTGGACACGGAGCTT-3’

MyoD1 - F: 5’-AGCGTCTCGAAGGCCTCAT-3’

R: 5’-AGCGCAGCTGAACAAGCTA-3’

Ki67 - F: 5’-TGGAAGAGCAGGTTAGCACTGT-3’

R: 5’-CAAACTTGGGCCTTGGCTGT-3’

### EdU incorporation analysis

EdU incorporation was performed in muscle fibers and cell cultures according to the manufacturer’s protocol (Invitrogen EdU assay Kit Catalog No. C10340).

### OPP incorporation assay

OPP incorporation was performed in C2C12 cells cultured for different conditions according to the manufacturer’s protocol (Invitrogen OPP assay Kit Catalog No. C10456). Cells were imaged using Leica SP8 TCS. Integrated fluorescence intensities were calculated using Cell Profiler. R was used to perform Multivariate ANOVA followed by Tukey’s HSD post hoc test, and ggplot was implemented for generating box and whisker plots. Estimating fluorescence intensities in MT involved approximations in ROI (boundary) selections for the purpose of quantification, as MT are multinucleated and overlapping.

### Apoptosis assay

To determine whether Fmrp knockdown cells undergo apoptosis, we used the Invitrogen Apoptosis Kit as per the manufacturer’s protocol (Cat no. V13245). Flow cytometry was performed on a BD Fortessa, using FlowJo software for analysis.

### Senescence assays

Control or Fmrp knockdown cells were evaluated for expression of p16 and p21 by qRT-PCR. Activity of senescence-associated β-galactosidase (β-Gal) enzyme was tested through X-gal staining assay at pH 6.0 to suppress lysosomal β-gal activity. Briefly, cells were fixed with 4% PFA for 10 min at room temperature, washed twice with PBS, and incubated with the chromogenic substrate 5-bromo-4-chloro-3-indolyl-beta-d-galactopyranoside (X-Gal) staining solution comprising 1 mg/ml X-gal (in DMF), 5 mM potassium ferrocyanide, 5 mM potassium ferricyanide and 1 mM MgCl_2_, overnight at 37 °C. DNA damage-induced foci of γH2AX were detected by immunofluorescence as detailed in the Supplementary Information.

### Polysome analysis

Fifteen million cells (MB, MT, or G0) were incubated with 0.1 mg/ml of Cycloheximide for 15 min or with 0.1 mg/mL of Puromycin for 2 h prior to lysis. Cells were lysed in ice-cold lysis buffer (10 mM Tris-Cl (pH 7.4), 150 mM KCl, 10 mM MgCl_2_, 1 mM DTT, 100 μg/ml Cycloheximide, 1% NP40, 1× cOmplete^TM^ EDTA-free Protease inhibitor (Cat. No. 5056489001), RNAse inhibitor (Cat. No. 10777-019), 6 U/ml). After incubation in cold lysis buffer for 30–45 min, the lysate was spun at 13000×*g* for 15 min at 4 °C. The supernatant was then loaded on to 10–45% (wt/wt) sucrose gradient (gradient buffer composition: 10 mM Tris-Cl (pH 7.4), 150 mM KCl, 10 mM MgCl_2_, 1 mM DTT, 100 μg/ml Cycloheximide) and samples were centrifuged at 39,000 rpm for 1.5 h at 4 °C in a SW41 Ti rotor (Beckman Coulter, Brea, CA, USA). The density-separated lysate was analyzed in a polysome profiler linked to a fraction collector (ISCO) with a UA-5 UV detector. Nine fractions of 1 ml were collected for each gradient and were used for either protein analysis by immunoblot or RNA analysis by qRT-PCR. IDAQ software was used for profile generation. The area under each ribosomal peak (40S, 60S, 80S, and polysomes) was calculated using Microsoft Excel and the average area of polysomes was divided by the average area of monosomes (80S ribosomes) for each profile in order to calculate polysome/monosome (P/M) ratio.

For isolation of RNA from polysome profiles [21, 35], we pooled fractions that constituted the mRNPs (fractions 1 & 2), monosomes (4 & 5), and polysomes (7 & 8) from each gradient derived from CHX- and Puro-treated MB, G0, and MT cells, and isolated RNA using TRIzol™ LS Reagent (Invitrogen) according to the manufacturer’s instructions. The isolated RNA was resuspended in equal volumes of water, quantified and checked for its purity by Nanodrop. Equal volumes of RNA from the pooled fractions were subjected to cDNA synthesis using SuperScript IV (Invitrogen) according to the manufacturer’s protocol. Quantitative real-time PCR analysis was performed using Power SYBR Green (Applied Biosystems) in ABI 7900HT Thermal cycler (Applied Biosystems). Serial dilutions of cDNA resulting in decrease in copy number were used to generate a standard curve for each gene. *C*_*t*_ values of each dilution were plotted against the copy numbers (ln of dilution). For experimental samples, the copy number was calculated using *C*_*t*_ value and standard curve obtained for that mRNA with the same set of primers. The percentage of total mRNA across the gradient was calculated as follows: (copy number in specific fraction/Total copy number of the gene across the gradient) × 100.

## Results

### mRNP components are differentially expressed in quiescent muscle stem cells versus myofibers

To investigate mRNP protein distribution in muscle, we used isolated murine myofibers e*x vivo*, complete with resident MuSCs in their niche. At a subcellular level, mRNP granule components are known to partition between a diffuse cytoplasmic distribution and punctate granules, where puncta represent functional complexes of RNA and proteins [36, 37]. Immunofluorescence analysis revealed that mRNP granule proteins are organized in puncta that were enriched in quiescent Pax7^+^ MuSC (Fig. 1). Myofibers also showed puncta, organized in striated patterns that suggest association with underlying cytoskeletal elements. Specifically, the translational repressor Fmrp showed punctate immunolabeling that was highly enriched in the cytoplasm of Pax7^**+**^ MuSC (Fig. 1B–D), but also associated with sarcomeres in myofibers (Fig. 1E–G), with distinct non-sarcomeric enrichment in the cytoplasm adjacent to Pax7^**-**^ myonuclei (Fig. 1C, C’). Interestingly, Fmrp was also located in MuSC nuclei (Fig. 1B, B’, D, D’), but not in myonuclei. Another translational repressor GW182, which is involved in the Ago-miRNA pathway, showed a similar distribution to Fmrp: discrete cytoplasmic puncta in Pax7^**+**^ MuSC and in zones near myonuclei, with smaller puncta in myofibers, arranged in a distinct pattern reflecting sarcomeric organization (Fig. 1 K). Thus, proteins implicated in translational repression are located in mRNP granules clearly evident in quiescent MuSC.

To determine the distribution of proteins involved in mRNA turnover, we examined expression of key regulators of mRNA, the decapping enhancer Dcp1a and the 5’-3’ exoribonuclease Xrn1 [14]. Dcp1a protein was not detected in quiescent MuSC (marked by MuSC-enriched membrane protein Caveolin 1 (Cav) (Fig. 1L), but formed a fine striated pattern in the myofiber cytoplasm, largely perpendicular to expected sarcomeric organization. Similarly, Xrn1 was not present in MuSC, but exhibited a clear striated pattern in myofibers (Fig. 1M). These observations reveal that components of the mRNA storage/stabilization complex (Fmrp, GW182) are highly expressed in the nuclei of quiescent MuSC, while the mRNA decay complex components (Dcp1a, Xrn1) are not.

### Reciprocal dynamics of Fmrp and Dcp1a puncta during MuSC activation on single fibers

To examine the dynamics of mRNP granules during MuSC activation, we cultured isolated myofibers for up to 48 h and determined the pattern of expression of Fmrp and Dcp1a. Activation of MuSCs led to EdU incorporation at 24 and 48 h. Fmrp puncta were prominent in quiescent MuSC, but showed dispersal and a diffuse staining pattern after 24 h. By contrast, Dcp1a puncta were not seen in quiescent MuSC, and became evident in activated and proliferating MuSC (EdU+) at 24 h (Fig. 2a–c). Thus, activation of MuSCs in their myofiber niche is associated with reciprocal changes in accumulation of mRNP granules associated with opposing functions, i.e., transcript turnover (Dcp1a) vs. utilization (Fmrp).

### Fmrp knockout mice exhibit altered muscle stem cell proliferation

**Fig. 3:**
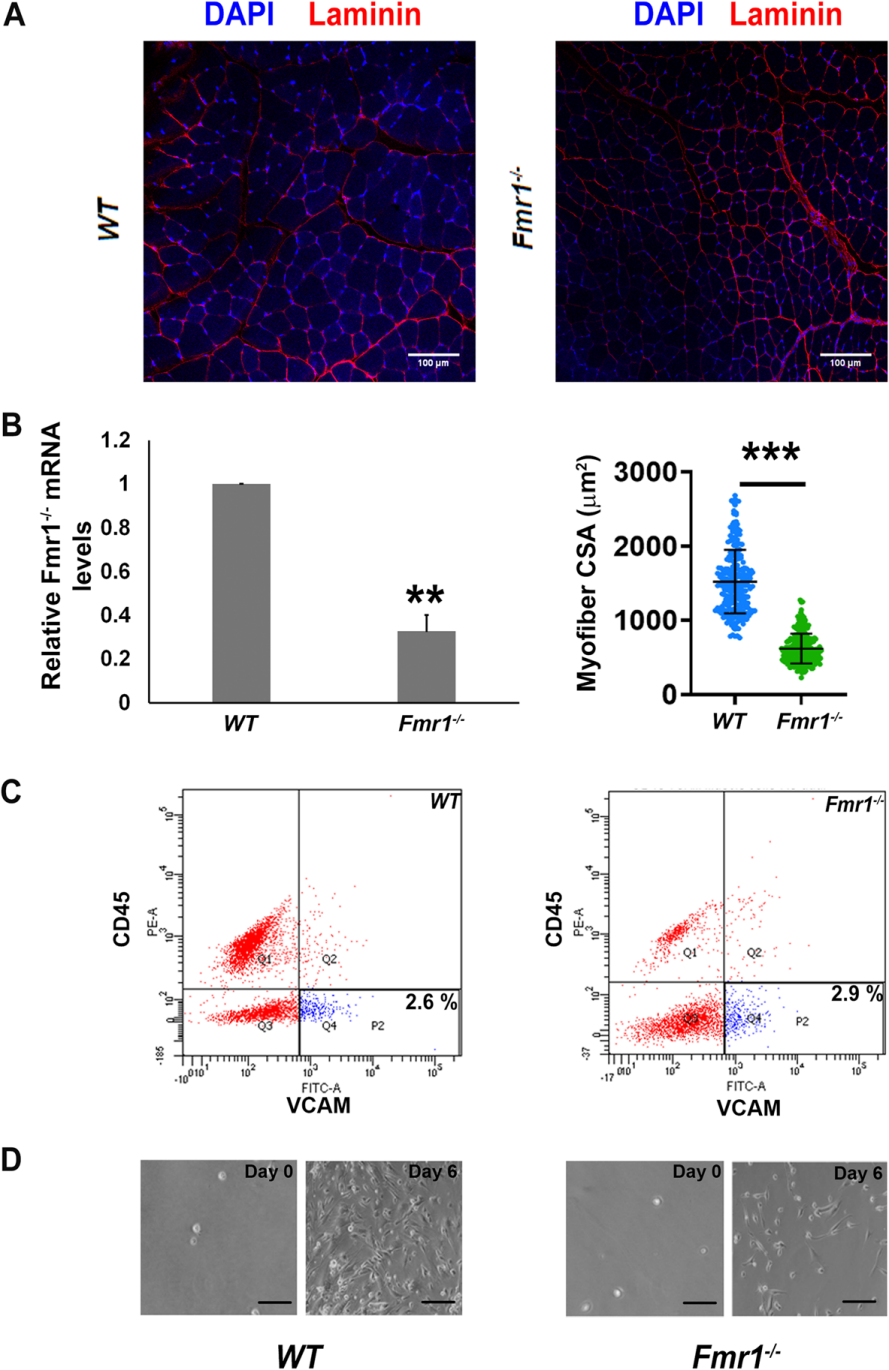
,Reduced muscle fiber caliber and MuSC proliferation in *Fmr1*^*-/-*^ mice. **a** Cryo-sections (20 μm) of adult tibialis anterior muscle isolated from wild-type (WT, left) and *Fmr1*^*-/-*^ mice (right) immunolabelled with laminin (red) and nuclei counterstained with DAPI (blue): myofibers show reduced diameter in *Fmr1*^*-/-*^ muscle. **b** Left panel shows qRT-PCR quantification of mRNA encoding *Fmrp* isolated from whole muscle of adult WT and *Fmr1*^*-/-*^ mice. Values represent mean + SEM; *n* = 2, two-tailed paired Student’s *t* test is indicated. ** *p* < 0.01. *Fmr1* RNA is detectable at lower levels in *Fmr1*^*-/-*^ as described [30, 38, 39]. Right panel: quantification of mean myofiber cross-sectional area (CSA) in wild-type and *Fmr1*^*-/-*^ muscle cryosections. Values represent mean + SD; *n* = 250. CSA from two mice. Two-tailed unpaired Student’s *t* test is indicated. *** *p* value < 0.0001, (*N* = 2 male mice per genotype). **c**–**d**. Muscle stem cells isolated from *Fmr1*^*-/-*^ mice do not proliferate well in culture. **c** The proportion of VCAM^**+**^, CD45^**-**^ MuSC is similar in adult WT and *Fmr1*^*-/-*^ mice. However, there is a noticeable reduction in the VCAM^**-**^**,** CD45^**+**^ cells suggesting effects on the leukocyte compartment. **d** Equal numbers of FACS purified MuSC isolated from the hind limb muscle of adult WT and *Fmr1*^*-/-*^ mice were plated in culture for 0 or 6 days. *Fmr1*^*-/-*^ cells show poor population expansion (*N* = 1 male mouse per genotype)

To examine whether Fmrp observed in mRNP granules in quiescent MuSC is important for stem cell function *in vivo*, we analyzed skeletal muscle from the *Fmr1* knockout (*Fmr1*^-/-^) mouse. Quantification of cross-sectional area of muscle fibers in cryo-sections of adult tibialis anterior muscle revealed that muscle fibers in *Fmr1*^-/-^ muscle showed drastically reduced caliber [mean ± SD of 619 μm^2^ ± 200] compared with age-matched wild-type (WT) mice [mean ± SD of 1518 μm^2^ ± 438, *n* = 250 fibers; *p* value < 0.0001] (Fig. 3a, b). As there is some expression of Fmrp in myofibers, a direct effect of this mRNP granule protein in a fiber-intrinsic mechanism cannot be ruled out. However, we also found that FACS-isolated *Fmr1*^-/-^ CD45^**-**^VCAM-1^**+**^ MuSCs (Fig. 3c) proliferate less compared to WT controls (Fig. 3d). The proportion of sorted MuSCs was similar in *Fmr1*^-/-^ and WT (Fig. 3c), by contrast to an earlier report by Fujita et al. [23], who reported that the number of Pax7^+^MyoD^-^ cells on single fibers were lower in *Fmr1*^-/-^ mice. This difference may reflect differences in the methods and markers used to identify MuSCs, as well as the numbers of cells analyzed in the two studies. Further, we found that when equal numbers of sorted WT and *Fmr1*^*-/-*^ MuSCs were plated, there were fewer *Fmr1*^-/-^ cells over the course of 6 days in culture compared to WT (Fig. 3d). Consistent with this observation, acute knockdown of Fmrp in C2C12 myoblasts reduced clonogenic performance (Fig. S3A) but did not lead to increased cell death through apoptosis (Fig. S3B), nor were markers of senescence significantly induced (Fig S4). Together, these preliminary results indicate that *Fmrp* expression is required for achieving normal fiber caliber in postnatal adult skeletal muscle and that this phenotype may be linked to a defect of knockout MuSC in proliferation, reactivation from quiescence or clonogenic self-renewal in culture.

### Differential expression of mRNP proteins in quiescent, proliferating, and differentiated muscle cells in culture

**Fig. 4:**
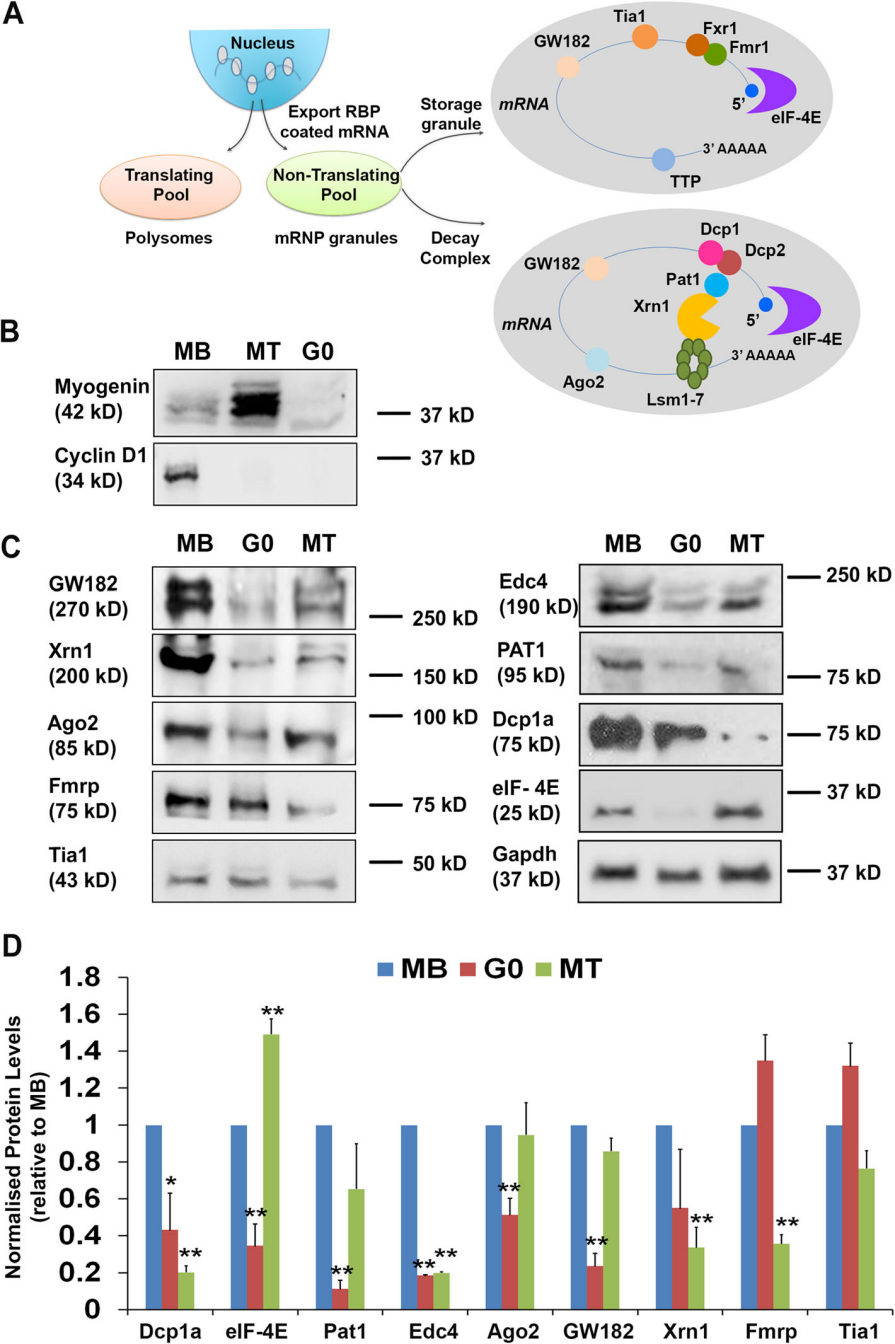
Differential expression of mRNP granule proteins in proliferating, quiescent and differentiated muscle cells in culture. **a** Schematic depicts segregation of transcripts into translating and non-translating pools on emergence from the nucleus with a constellation of RNA-binding proteins. Non-translated transcripts may be sequestered in mRNPs enriched for decay complex (mRNA turnover) or storage granule components (translational repression/stabilization of mRNA). **b** Western blot analysis showing that three distinct cellular states can be distinguished by expression of Myogenin and Cyclin D1 (MB: asynchronously proliferating myoblasts are CycD1+, MyoG-; MT: 5-day differentiated myotubes are CycD1-, MyoG+; G0: quiescent myoblasts are CycD1-, MyoG-). **c** Western blot profile of mRNP granule protein expression across three cellular states. MB: proliferating myoblasts; G0: quiescent myoblasts; MT: differentiated myotubes. Expression of most proteins is suppressed in G0; notable exceptions are Fmrp and Tia1, which are maintained in G0 (see Table 1 and quantification in Fig. 4d). **d** Quantification of relative expression of mRNP granule proteins across the three cellular states calculated from densitometric analysis of immuno-blots. Each protein was normalized to Gapdh in the same sample, before normalizing to MB. Values represent mean + SD, *n* = 3, two-tailed paired Student’s *t* tests are indicated as [* *p* ≤ 0.05, ** *p* ≤ 0.01]

,To explore the muscle cell-intrinsic functions of Fmrp, we examined the expression of a series of mRNP proteins (schematized in Fig. 4a), in a tractable adult MuSC-derived murine C2C12 culture model that permits the generation of pure populations of proliferating myoblasts (MB), quiescent myoblasts (G0), or differentiated myotubes (MT) [2, 26]. In particular, this model allows the entry to mitotic quiescence to be examined, a limitation in other similar techniques. The three cellular states were distinguished using expression of Myogenin (Myog), a master regulator of myogenic differentiation, and Cyclin D1, a canonical marker of proliferation: MB are Cyclin D1^**+**^ Myog^**-**^, MT are Cyclin D1^-^ Myog^+^ and G0 are Cyclin D1^-^ Myog^-^ (Figs 4b and S5A). We investigated the abundance of Fmrp and Dcp1a, together with other categories of mRNP components, namely: (i) proteins involved in translation repression and formation of SGs (Fmrp, Tia1) [10, 40], and translation initiation (eIF-4E), (ii) proteins involved in the PB nonsense-mediated mRNA decay pathway (Dcp1a, Pat1, and Edc4), and (iii) proteins known to shuttle between these two complexes, (Xrn1, Gw182, and Ago2) [10, 41] (Fig. 4c, d). Briefly, the translation repressors Fmrp and Tia1 continued to be expressed in G0 at levels similar to MB, but in MT, Fmrp was downregulated. Consistent with reversible suppression of translation in G0, eIF-4E, the cap-binding component of the rate limiting translation initiator eIF-4F complex, is strongly downregulated in G0, but upregulated in MT. Dcp1a (and another mRNA decay factor Edc4) were less abundant in both G0 and MT compared with MB. Overall, the quantitative analysis of mRNP granule protein expression (Fig. 4c, d) revealed that when comparing G0 to MB, proteins involved in mRNA turnover such as Dcp1a and Edc4, were under-represented, while proteins involved in mRNA storage/stabilization/translational stalling (Fmrp, Tia1) were maintained at similar levels in G0 when compared to MB.

To assess whether changes in expression of mRNP proteins resulted from changes in expression of their mRNAs, we used bioinformatic analysis of available transcriptome data from *in vivo* fixed (quiescent) satellite cells compared with activated (proliferating) satellite cells [42] and grouped them according to their function [43, 44]. This comparison revealed that expression of genes encoding translational stalling complex proteins (Tia1, Tncr6b, Ddx5, Ddx17) are upregulated in G0, whereas genes encoding proteins important for mRNA decapping and turnover (Dcp1a, Edc4, Edc3) are downregulated in G0 (Table 1). Fmrp expression was found to be maintained in quiescent satellite cells. Taken together, this analysis suggests that compared with proliferating MB, the decapping machinery is suppressed in G0, but that translational repression capacity is maintained/enhanced in G0.

**Table 1:** Bio-informatic analysis of transcripts encoding mRNP components

Data from Yue et al (2020)	Accession	GSE113631						
mRNP component genes Table 1		FPKM values				fSC vs ASC		
Functional Class	Gene Symbol	ASC_rep1	ASC_rep2	fSC_rep1	fSC_rep2	Fold change (Log2)	adjusted p-value (BH correction)	Fold change
ARE binding	Tnpo1	57.211	65.710	17.063	15.135	-1.93	0.0353	0.26
ARE binding	Zfp36	22.033	19.558	15.694	13.194	-0.53	0.1265	0.69
ARE binding	**Tia1**	7.034	6.693	16.816	16.464	1.28	0.0117	2.42
Deadenylation	Cnot1	105.514	110.661	20.399	21.317	-2.37	0.0135	0.19
Deadenylation	Cnot2	41.603	42.019	26.262	15.837	-0.99	0.1112	0.50
Deadenylation	Cnot6l	32.610	34.390	24.860	25.916	-0.4	0.0481	0.76
Deadenylation	Cnot8	25.708	26.029	30.524	23.093	0.05	0.8439	1.04
Deadenylation	Pan2	12.528	10.940	15.276	14.178	0.33	0.1543	1.26
Deadenylation	Tob2	11.102	12.033	16.704	14.161	0.42	0.1717	1.34
Deadenylation	Cnot4	10.540	8.787	11.628	17.230	0.58	0.3379	1.49
Deadenylation	Pan3	3.337	3.622	5.453	5.373	0.64	0.0281	1.56
Deadenylation	Cnot3	0.864	0.889	3.501	3.168	1.93	0.0252	3.81
Decapping	Lsm3	249.390	284.670	85.416	78.982	-1.7	0.0357	0.31
Decapping	**Dcp1a**	16.651	17.486	5.447	5.466	-1.65	0.0151	0.32
Decapping	Edc3	12.841	12.809	5.918	4.803	-1.26	0.0274	0.42
Decapping	Patl1	10.775	9.751	5.821	4.656	-0.97	0.0601	0.51
Decapping	**Edc4**	24.210	23.716	12.896	12.523	-0.91	0.0130	0.53
Decapping	Dcp2	9.174	10.101	7.586	8.180	-0.29	0.1489	0.82
Decapping	Lsm2	32.104	34.063	28.098	31.387	-0.15	0.3121	0.90
Decapping	Lsm6	209.767	214.354	204.279	179.065	-0.15	0.3451	0.90
Decapping	Lsm7	58.943	65.467	66.429	58.668	0.01	0.9582	1.01
Decapping	Lsm5	87.274	87.571	126.064	111.808	0.44	0.0971	1.36
Decapping	Lsm1	4.656	4.518	7.664	5.746	0.55	0.2374	1.46
Decapping	Dcp1b	0.440	0.394	2.564	2.611	2.63	0.0081	6.20
Decapping	Lsm4	5.825	5.767	42.218	38.352	2.80	0.0207	6.95
Many RNP functions	Eif4e	230.579	234.878	57.512	38.165	-2.28	0.0202	0.21
Many RNP functions	Ddx1	56.948	56.634	16.101	9.526	-2.15	0.0276	0.23
Many RNP functions	Eif2s2	183.202	191.179	68.677	56.697	-1.58	0.0214	0.33
Many RNP functions	Eif2b1	76.122	77.151	33.579	23.721	-1.42	0.0379	0.37
Many RNP functions	Gemin5	70.000	66.713	28.690	30.406	-1.21	0.0184	0.43
Many RNP functions	Trim59	4.600	4.463	2.249	1.956	-1.11	0.0247	0.46
Many RNP functions	Lima1	15.817	12.996	8.282	10.047	-0.65	0.1513	0.64
Many RNP functions	Dhx40	5.881	6.893	5.668	4.463	-0.33	0.3256	0.79
Many RNP functions	Pdlim7	15.903	17.440	19.606	18.779	0.20	0.1690	1.15
Many RNP functions	Fmr1	42.281	42.132	58.735	49.227	0.35	0.2056	1.28
Many RNP functions	Xrn1	11.279	11.434	14.211	17.589	0.49	0.1853	1.40
Many RNP functions	Eif2b4	22.085	21.683	33.687	31.827	0.58	0.0322	1.50
Many RNP functions	Pcbp2	77.519	73.659	138.630	159.083	0.98	0.0542	1.97
Many RNP functions	Trim28	19.447	19.682	33.020	45.418	1.00	0.1500	2.00
Many RNP functions	Lpp	30.789	32.101	113.147	97.692	1.75	0.0385	3.35
Many RNP functions	Ddx5	65.868	58.928	263.633	201.650	1.90	0.0746	3.73
Many RNP functions	Peg3	105.426	104.905	445.266	466.449	2.12	0.0135	4.33
Many RNP functions	Ybx1	64.225	64.993	464.325	296.992	2.56	0.1191	5.90
Many RNP functions	Ddx17	9.400	8.959	111.803	114.915	3.63	0.0081	12.35
Many RNP functions	Trim21	0.015	0.017	0.417	0.613	5	0.0809	32.00
miRNA-mediated gene silencing	Limd1	46.125	42.266	43.565	43.929	-0.01	0.8565	0.99
miRNA-mediated gene silencing	Tnrc6a	8.243	8.496	10.950	13.634	0.55	0.1674	1.47
miRNA-mediated gene silencing	Htt	2.976	2.676	6.126	6.307	1.14	0.0195	2.20
miRNA-mediated gene silencing	**Tnrc6b**	4.196	4.958	14.202	15.669	1.71	0.0293	3.27
miRNA-mediated gene silencing	Ipo8	4.524	4.272	16.861	15.367	1.87	0.0242	3.66
miRNA-mediated gene silencing	Tnrc6c	3.082	3.039	16.137	17.995	2.48	0.0247	5.58
NMD pathway	Smg7	58.640	66.881	11.867	12.560	-2.36	0.0299	0.19
NMD pathway	Upf2	13.025	15.822	16.257	16.691	0.19	0.3801	1.14
NMD pathway	Upf1	14.745	16.093	17.029	20.119	0.27	0.2885	1.21
NMD pathway	Pnrc2	44.583	42.606	61.898	49.546	0.35	0.2770	1.28
NMD pathway	Upf3b	24.102	22.611	42.912	44.988	0.91	0.0230	1.88
NMD pathway	Smg5	2.169	2.287	7.059	6.539	1.61	0.0217	3.05
NMD pathway	Smg6	6.228	6.373	26.228	27.657	2.10	0.0148	4.28
NMD pathway	Upf3a	5.788	5.261	34.781	29.334	2.54	0.0379	5.82
NMD pathway	Pnrc1	3.468	2.819	103.360	88.526	4.93	0.0293	30.52

### Distinct organization and dynamics of mRNP granules in two mitotically inactive states

**Fig. 5:**
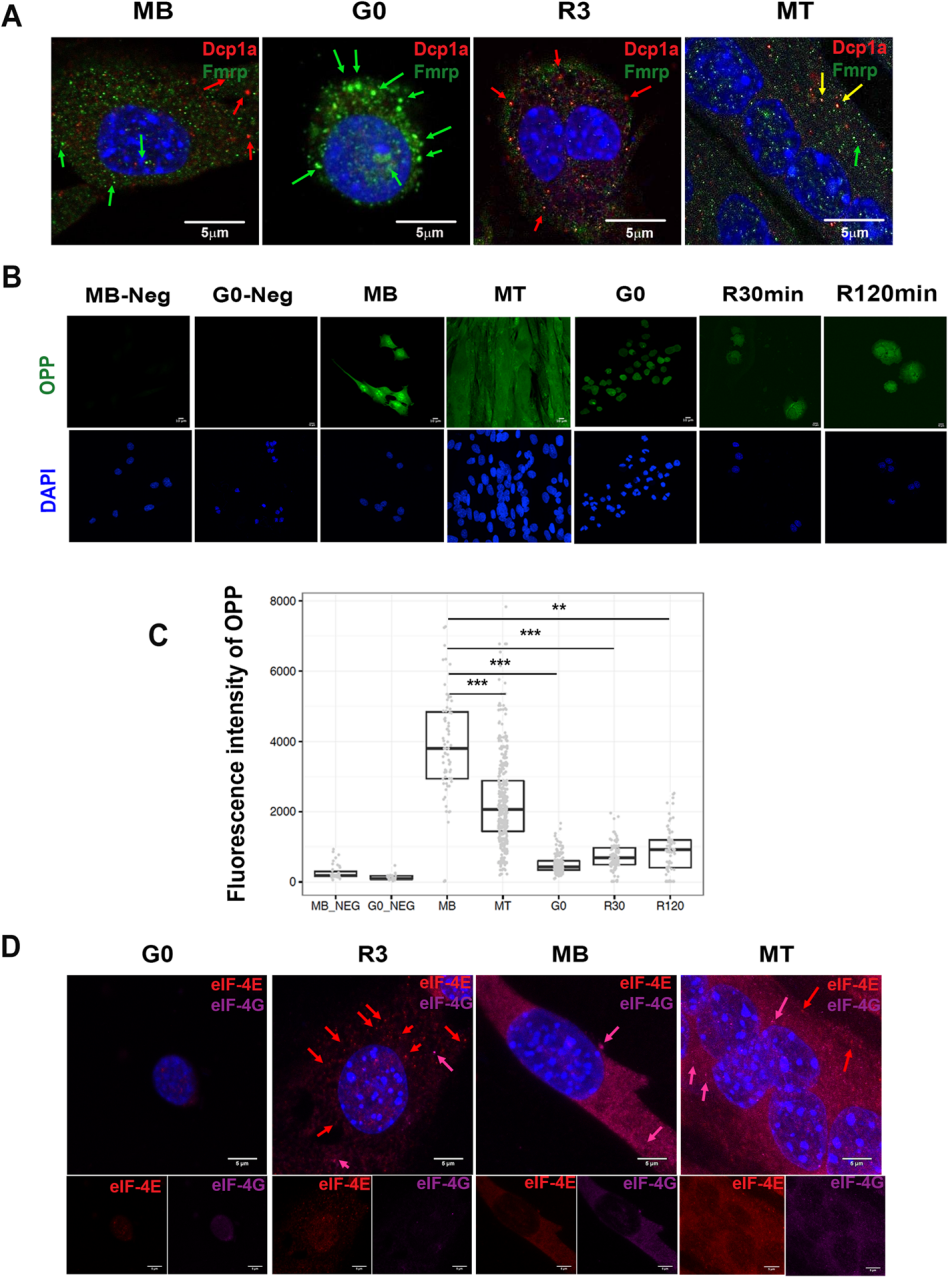
Assembly of mRNP into puncta in different cellular states correlates with levels of protein synthesis. **a** Representative immunofluorescence images showing Fmrp (green) and Dcp1a (red) puncta in G0, MB, and MT, as well as cells reactivated for 3 h from G0 (R3). Arrows indicate prominent puncta. Notably, Fmrp puncta are large and prominent in G0, disperse at 3 h post reactivation and are less evident in asynchronous MB. Dcp1a puncta are nearly absent in G0 and reappear at R3; Dcp1 puncta are also more prominent in MB than MT. Fmrp and Dcp1a mostly localize to distinct puncta; the rare yellow puncta seen in R3 and MT may reflect transient co-localization due to passage of transcripts between two types of mRNP granules as inferred in [45]. **b** Measurement of the rate of protein synthesis using OPP incorporation into newly synthesized proteins reveals active translation in MB and MT, and substantial suppression in G0. **c** Quantification of images in **b**. Fluorescent intensity was measured in 150 cells from each condition, the box and whisker plot shows integrated fluorescence for each cell (each dot represents one cell), limits on the box correspond to 75th and 25th percentile values. “Mb-neg” and “G0-neg” represent samples of MB and G0 that were not pulsed with OPP but processed for detection along with samples that were exposed to OPP. *N* = 2 biological replicates. Data were analyzed by multivariate ANOVA with post hoc HSD Tukey tests performed for each pairwise comparison. *** *p* < 0.0001, ** *p* < 0.001. **d** Immunolabeling of translation initiation factors eIF-4E (red) and eIF-4G (pink) in G0, R3, MB, and MT: Upper panel shows merged images, and lower panels show detection of each factor individually. Expression and assembly of these translation factors correlates with levels of protein synthesis seen in **b** and **c**: poor in G0, restored assembly with distinct puncta in R3, and strong expression and organization of eIF-4E and eIF-4G complexes in MB and MT

To compare the distribution and dynamics of mRNP complexes in different cellular states in culture, we examined the staining pattern of Fmrp and Dcp1a using immunofluorescence confocal microscopy (Fig. 5a). As active mRNPs self-assemble into observable puncta and disassemble upon releasing bound mRNA [36, 43], subcellular staining patterns are a reflection of the activity state of these complexes. In asynchronously proliferating MB, Dcp1a and Fmrp were present in small, numerous, non-overlapping cytoplasmic puncta, consistent with their participation in distinct complexes with distinct functions. In G0, whereas Dcp1a immunolabeling was low and diffuse (not punctate), the size and intensity of cytoplasmic Fmrp granules dramatically increased, and nuclear-localized Fmrp granules were also prominent, while total Fmrp protein level was maintained (Fig. 4c, d), suggesting enhanced granule assembly, and greater translational repression. Notably, mRNP immuno-detection patterns in cultured G0 cells (Fig. 5a) reflected the patterns observed *in vivo* in MuSC (Fig. 1) with respect to (i) increased Fmrp puncta and reduced Dcp1a puncta and (ii) the appearance of Fmrp puncta in the G0 nucleus.

,We next tested the effect of cell-cycle reactivation on mRNP granules. Three hours after reactivation from quiescence (R3) Fmrp puncta disappeared and Dcp1a puncta re-appeared, consistent with the abundance of Dcp1a protein in cycling MB (Fig. 5a). In MT, Fmrp was organized as small, dispersed cytoplasmic granules, while Dcp1a puncta were reduced compared to MB (Fig. 5a). There was a general similarity in abundance of puncta in culture and *in vivo*: i.e., similar patterns in MT and myofiber versus G0 and MuSC, suggesting an association of particular mRNP granule dynamics with these distinct cellular states. The degree to which three additional mRNP proteins (Edc4, Pat1, Ago2) were organized into puncta also varied between cellular states (Fig. S1). Additional representative images (Fig. S2A) show the increased presence of Fmrp puncta in G0 and Dcp1a puncta in proliferation, which is supported by quantification of mean fluorescence intensities of these proteins in subcellular puncta (Fig. S2B), and confirms the reciprocal expression/organization of Fmrp and Dcp1a in G0 compared to proliferating cells. Taken together, these immuno-localization studies indicate that translational repression complexes (Fmrp, Ago2) are more prominent in G0 than in MB and MT, and that nonsense-mediated mRNA decay complexes (Dcp1a, Edc4, Pat1) are more prominent in MB and MT than in G0, both of which are consistent with earlier reports of transcript stabilization in quiescent cells [32, 34, 46].

### Global translation rates and expression of translation initiation factors are suppressed in G0

To compare global translation rates between the proliferating, differentiated and quiescent states, we used incorporation of O-propargyl-puromycin (OPP) to biosynthetically label nascent proteins (Fig. 5b, c). Rapidly growing MB pulsed with OPP for 1.5 h showed strongly labeled cytoplasm and nucleoli, possibly reflecting the nucleolar location of newly synthesized ribosomal proteins during ribosome biogenesis. In fused MT, cytoplasmic OPP labeling predominated, likely reflecting greater synthesis of sarcomeric and other non-ribosomal proteins. By contrast, G0 cells showed low and variable OPP labeling of the cytoplasm. Many G0 cells were essentially unlabeled above background levels (Fig. 5b, c). Moreover, nucleoli could not be distinguished (Fig. 5b). These findings indicate lower rates of protein synthesis and ribosome assembly in G0 cells.

To investigate translation by an independent method, we analyzed expression of two translation initiation factors, eIF-4E (the rate limiting factor in cap-dependent translation that also regulates mRNA export) and eIF4G (a scaffold for assembly of the eIF-4F complex comprising eIF-4E, eIF-4G, and eIF-4A on the 5’ cap). Both proteins were present in MB and MT, but both were much reduced in G0 (Fig. 5d), and strongly re-induced in a punctate pattern after reactivation from quiescence (R3), when protein synthesis begins to recover (Fig. 5 c, d). The altered abundance of eIF-4E was consistent with our western analysis (Fig. 4 c, d). These initiation factors showed some nuclear localization in G0, which was greatly enhanced during synchronous reactivation, but not detected in either cycling MB or MT, possibly reflecting involvement in upstream functions such as mRNA export, that are important for cell cycle re-entry (Fig. 5d). Taken together, these findings are consistent with the notion of G0 as a state where global translational repression is coupled to mRNA stabilization in granules, keeping cells primed for cell cycle re-entry [17, 34].

### Quiescent myoblasts exhibit puromycin-resistant mRNP complexes in G0

**Fig. 6:**
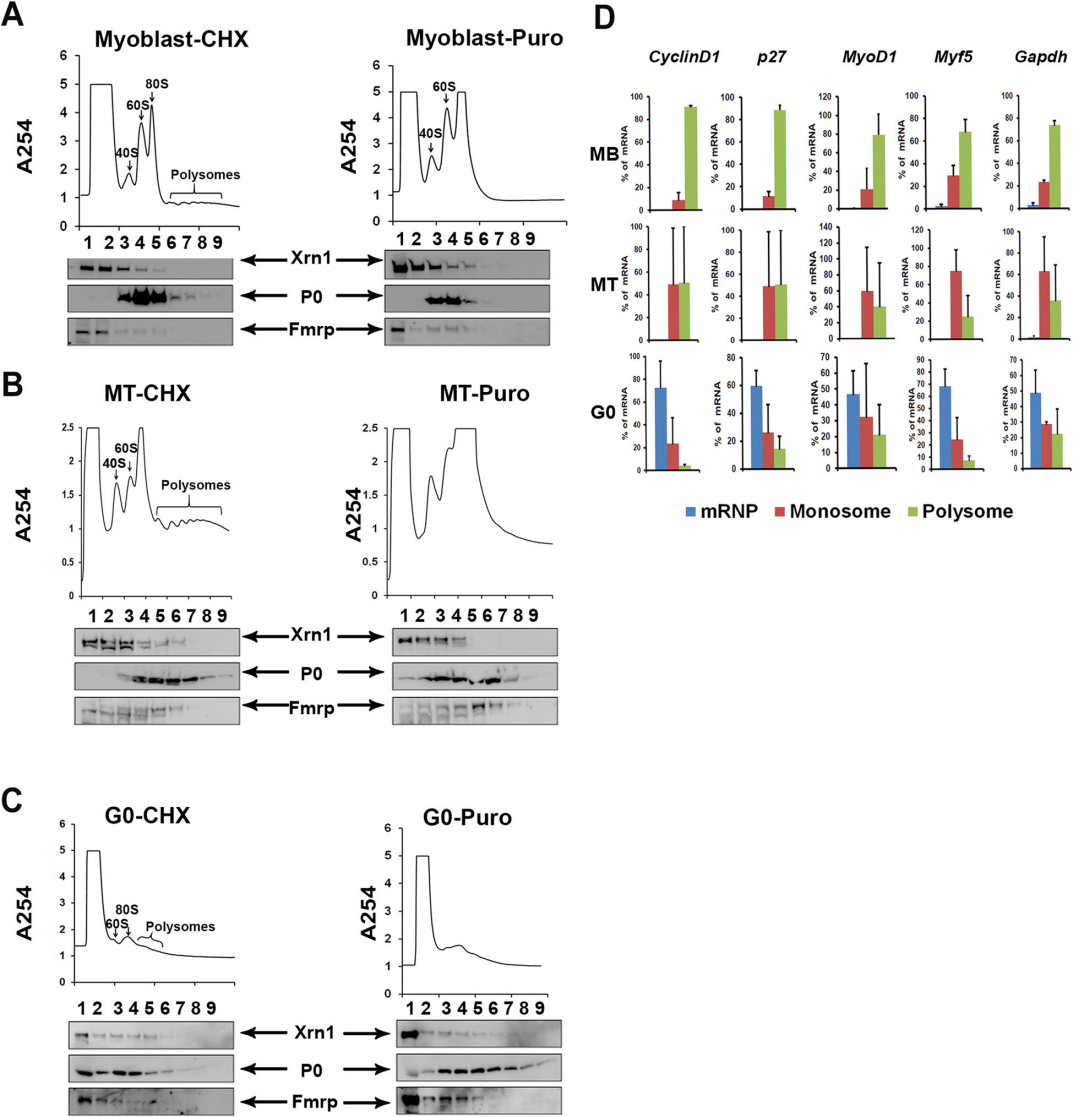
Polysome profiles of proliferating, quiescent, and differentiated muscle cells reveal stalled polysomes in G0. Translational profiles of myoblasts (**a**), myotubes (**b**), and G0 cells (**c**) using polysome display on sucrose gradients. Panels on left depict profiles derived from cells briefly treated with CHX to ‘fix’ ribosomes in the act of translation, while panels on the right depict profiles derived from cells treated with Puro to disrupt translation by mRNA release. Western blotting of proteins isolated from 9 individual 1-ml fractions from the sucrose gradients (equal volumes loaded) reveals (i) distribution of ribosomes in each fraction based on ribosomal protein P0 (middle) and the extent of association of decay complex based on Xrn1 (top), and translation inhibitory complex based on Fmrp (botttom) with each fraction. Comparison of the profiles and distribution of individual proteins reveals very poor translation in G0, correlating with the OPP incorporation in Fig. 5. The presence of puromycin-insensitive complexes in G0 arrested cells, suggests polysome stalling. **d** Analysis of transcript distribution in polysome profiles correlates with rate of protein synthesis and suggests low mRNA utilization in G0. qRT-PCR analysis of selected transcripts (*GAPDH, Cyclin D1, MyoD, Myf5,* and *p27*) from RNA isolated from the mRNP-, monosome-, and polysome-containing fractions of profiles depicted in Fig. 6a–c. All transcripts tested show substantial enrichment in the mRNP and monosome compartment in G0 compared with the monosome and polysome fraction, suggesting a severe suppression of protein synthesis consistent with the OPP incorporation study (Fig. 5b, c). Notably, none of the transcripts tested show appreciable enrichment in the mRNP fraction in MB and MT, indicating their robust translational utilization in the polysomal compartment. Values represent the mean + SD of transcript levels in fractions from two independent polysome profiles for each condition

,Quiescent cells show low transcriptional activity compared with proliferative or differentiated states. Although many transcripts are specifically induced in G0 [1, 47] and some must be translated into proteins required for the maintenance of quiescence [48], the data above suggest that a number of G0-induced transcripts may also be sequestered in non-polysomal compartments, to be mobilized for protein synthesis required for the return to the cell cycle [17]. To visualize ongoing translation activity directly, we analyzed steady-state polysome profiles in each cellular state. To ensure polysome integrity during isolation and display, cells were treated briefly with cycloheximide (CHX) prior to lysis, to arrest translating ribosomes on mRNAs, followed by separation on sucrose density gradients (Fig. 6a–c). The profile of RNA-protein complexes was quantified in density-separated fractions and analyzed by immuno-blotting. A second profile was run from cells in each state that were treated briefly with puromycin (Puro), that successfully disengaged mRNA from translating ribosomes, removing the polysome profile (Fig. 6a–c). With respect to mRNP granule dynamics, CHX rapidly dissociates mRNP granules, whereas Puro promotes their assembly [10].

In MB, in addition to the strong ribosomal subunit peaks in fractions 3, 4, 5 (containing free 40S, 60S subunits, and 80S monosomes, respectively), a range of polysome peaks was visible (fractions 6–9), which was sensitive to Puro treatment, showing that the cells were engaged in active translation (Fig. 6a). Western blot profiles confirmed that the ribosome-containing heavier fractions (3–9, marked by the presence of the ribosomal protein P0) were largely devoid of mRNP proteins Xrn1 and Fmrp, which were enriched in the non-polysomal fractions 1–2. On treatment with Puro, disruption of polysomes was evident and accompanied by loss of P0 protein from fractions 6–9 (Fig. 6a). MT also displayed very active translation, showing monosome and polysome peaks similar to the profile in MB (Fig. 6b). Similarly, Xrn1 and Fmrp were detected at low levels in fraction 6, but otherwise, these mRNP proteins were largely absent from the polysome fractions 7–9. As in proliferating MB, Puro treatment led to the loss of polysome peaks in MT.

In G0 cells, by contrast, polysomes were nearly undetectable and fewer monosomes were seen, consistent with the accumulation of P0 in the lighter complexes (Fractions 1–4 (Fig. 6c). Nevertheless, in G0 cells, P0 persisted in high molecular weight complexes (Fractions 6–9 in Puro vs CHX), that were insensitive to Puro (Fig. 6c). Together, these observations suggest the presence of heavy mRNP complexes in G0 cells that are not engaged in active translation. These heavy mRNPs could be stalled polysomes, or mRNA captured in other heterogeneous paused complexes along with ribosomes, but not undergoing active translation. Treatment of G0 cells with Puro increased mRNPs in the heavy fractions 7–9, the opposite of the effect of Puro in MB (Fig. 6a, c). The sustained enrichment in the heavier fractions (7–9) in puromycin-treated G0 cells suggests that ribosomal proteins are present in non-canonical high molecular weight complexes in G0 cells, which are absent in MB and MT. Taken together with OPP incorporation and eIF-4E expression levels, these results demonstrate that proliferating and differentiated cells are actively engaged in translation, while quiescent cells show markedly suppressed protein synthesis, potentially associated with sequestered and stalled ribosomes.

### Transcripts accumulate in a non-polysomal mRNP compartment specifically in G0

To probe the distribution of specific transcripts between actively translating and inactive sequestered compartments, we used qRT-PCR analysis on RNA isolated from the mRNP, monosome- and polysome-containing fractions (Fig. 6d). We selected mRNAs whose levels are (i) unchanged (*Gapdh*) (ii) suppressed in G0 (*Cyclin D1*, *MyoD*), or (iii) maintained/induced in G0 (*Myf5*, *Cdkn1b*/*p27*), as seen in global transcript analysis derived from transcriptome data (Venugopal et al, 2020 [49]) (Fig. S5B and Table 2). Consistent with the bulk polysome profile that shows low polysome assembly in G0, all transcripts tested show substantial enrichment in mRNP and monosome compartments and < 10% in the polysome fraction in G0 cells (Fig. 6d). By contrast, in both MB and MT, all five mRNAs were enriched on polysomes, with barely detectable presence in the mRNP fraction, with Fig S5B reflecting the global transcript status of the mRNAs relative to MB consistent with the high rates of protein synthesis typical of these states. Thus, the observation that, for all five transcripts tested, the majority (as a proportion of each transcript’s abundance in each state), was either found in large polysomes in MB (i.e., was actively translated), shifted to monosomes compared with polysomes in MT (i.e., was less actively translated), or further shifted to mRNPs in cells in G0 (i.e., was not translated at all), we interpret to reflect the global translational status of each state. Taken together with the repressed global rates of protein synthesis and increased accumulation of mRNP proteins in visible puncta, we conclude that mRNA sequestration in a non-translated compartment is a broad regulatory process that is enhanced in reversible G0, but not in post-mitotic MT.

**Table 2:** Non-translating to translating ratio (mRNP: mono + poly) ratio in 3 different states (MB, MT, and G0)

State	Transcript				
	*Cyclin D1*	*p27*	*MyoD*	*Myf5*	*Gapdh*
MB	0:100	0:100	0:100	2:98	3:97
MT	0:100	0:100	0:100	0:100	0:100
G0	70:30	60:40	50:50	65:35	50:50

### Dcp1a and Fmrp reciprocally regulate their protein abundance and granule assembly

**Fig. 7:**
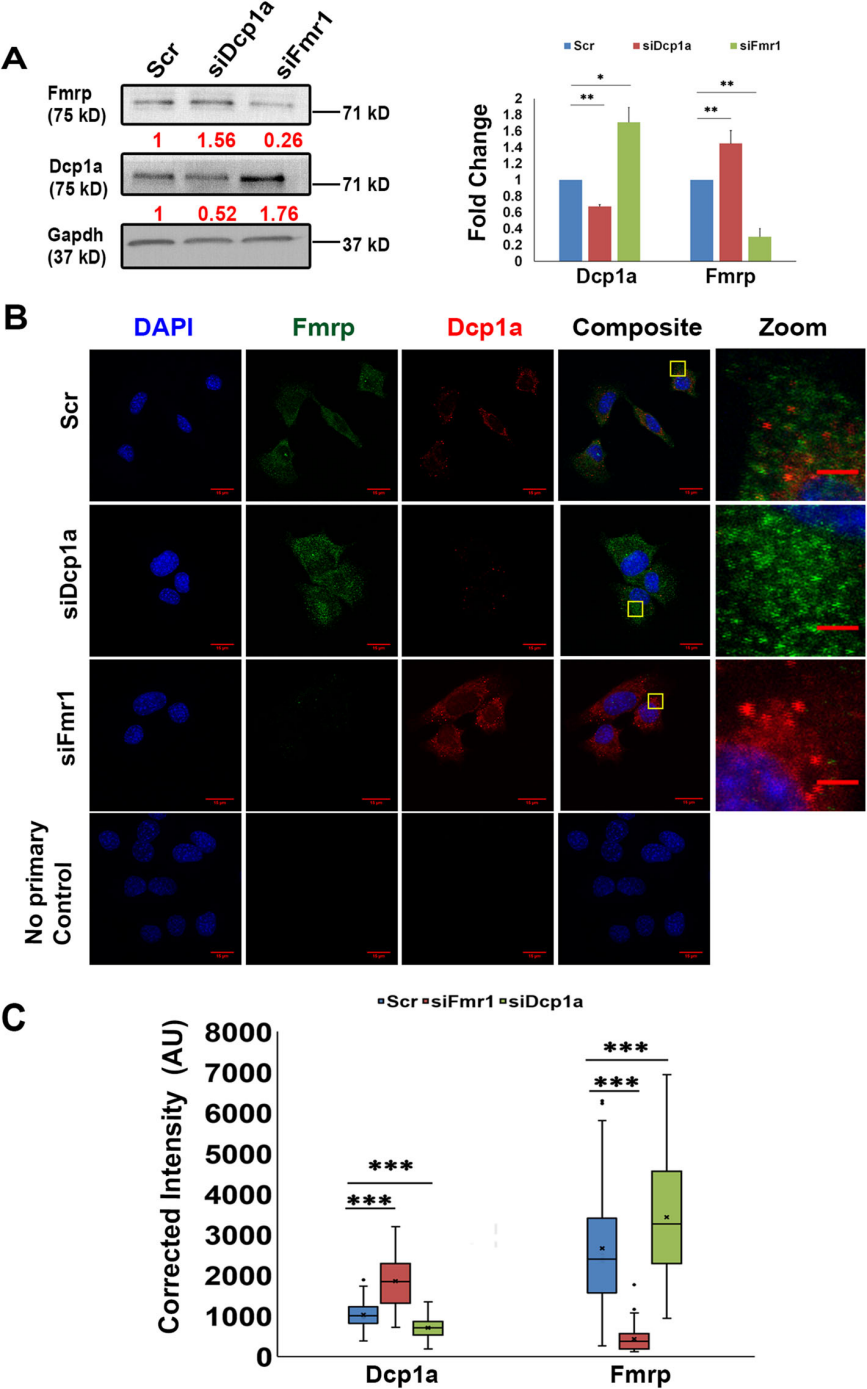
Cross-regulation of Fmrp and Dcp1a in knockdown myoblasts. **a** Following transfection with either Dcp1a or Fmr1 siRNA pools or the control pool (Scr), knockdown myoblasts were incubated in growth medium for 18 h. Left: Immunoblot analysis shows that in Fmrp knockdown, Fmrp abundance is reduced but Dcp1a expression is enhanced. Likewise, Fmrp protein levels are increased in Dcp1a knockdown. Values depicted under each lane represent protein levels from normalized densitometric scans, relative to level in Scr. Right panel: Densitometry of western blots of Dcp1a, and Fmrp proteins normalized relative to Scr with Gapdh as internal control. Bar graphs represent mean ± SD from *n* = 3. Two-tailed paired Student’s *t* tests are indicated as * *p* < 0.05. ** *p* < 0.01. **b** Knockdown effects on Dcp1a and Fmrp by their respective targeting siRNAs are detectable at the subcellular level. Consistent with changes at the level of protein abundance, immunofluorescence analysis shows that increased Dcp1a expression in Fmr1 knockdown is accompanied by enhanced Dcp1a puncta assembly, while compromising Dcp1a expression leads to enhanced Fmrp puncta assembly. Scale bars represent 15 μm except in zoomed panels where scale bars represent 8 μm. **c** Quantitative image analysis of **b**: The fluorescence intensities of Fmrp and Dcp1a following immunostaining of Scr, siFmr1, and siDcp1a samples were calculated and represented as box and whisker plots. Significant increases in Dcp1a puncta were observed in Fmrp knockdown and vice versa increased Fmrp puncta were observed in Dcp1a knockdown. Data were obtained from triplicate samples for each condition and graph shows scoring of at least *n* = 75 cells each for Scr (Scrambled), siFmr1 (Fmrp knockdown), siDcp1a (Dcp1a knockdown). Two-tailed paired Student’s *t* test results are indicated as *** *p* < 0.001

As Fmrp and Dcp1a are known to regulate distinct aspects of mRNA function (translation vs. turnover [15]) and were found in different complexes, we considered the possibility that these proteins might also cross-regulate. We used siRNA-mediated knockdown to perturb the levels of each protein and evaluated the effect of knockdown of one protein on abundance of the other protein using western blotting (Fig. 7a). Proliferating myoblasts were transfected with siRNA smart pools (comprising four independent siRNAs) designed to target either *Dcp1a* or *Fmr1* mRNAs. A non-targeting siRNA pool was used as a control. Knockdown efficiency was confirmed to be 70–85% for Fmrp and 40–50% for Dcp1a by western blotting (Fig. 7a). Indeed, knockdown of Fmrp led to an induction of Dcp1a protein levels and vice versa, knockdown of Dcp1a was accompanied by higher levels of Fmrp (Fig. 7a). This reciprocal regulation at the level of protein abundance was accompanied by increased detection of the respective protein in cytoplasmic puncta (Fig. 7b). Quantification of the fluorescent intensity of cytoplasmic staining (Fig. 7c) revealed that knockdown of Fmrp was readily observed as reduced immunofluorescence, and accompanied by an enhanced intensity of Dcp1a, and reciprocally, knockdown of Dcp1a, led to loss of Dcp1a detection and enhanced intensity of Fmrp. Taken together, these experiments reveal cross-regulation of Fmrp and Dcp1a not only at the level of protein abundance, but also at the level of protein assembly into puncta.

### Fmrp and Dcp1a play opposing roles in control of MB proliferation

**Fig. 8:**
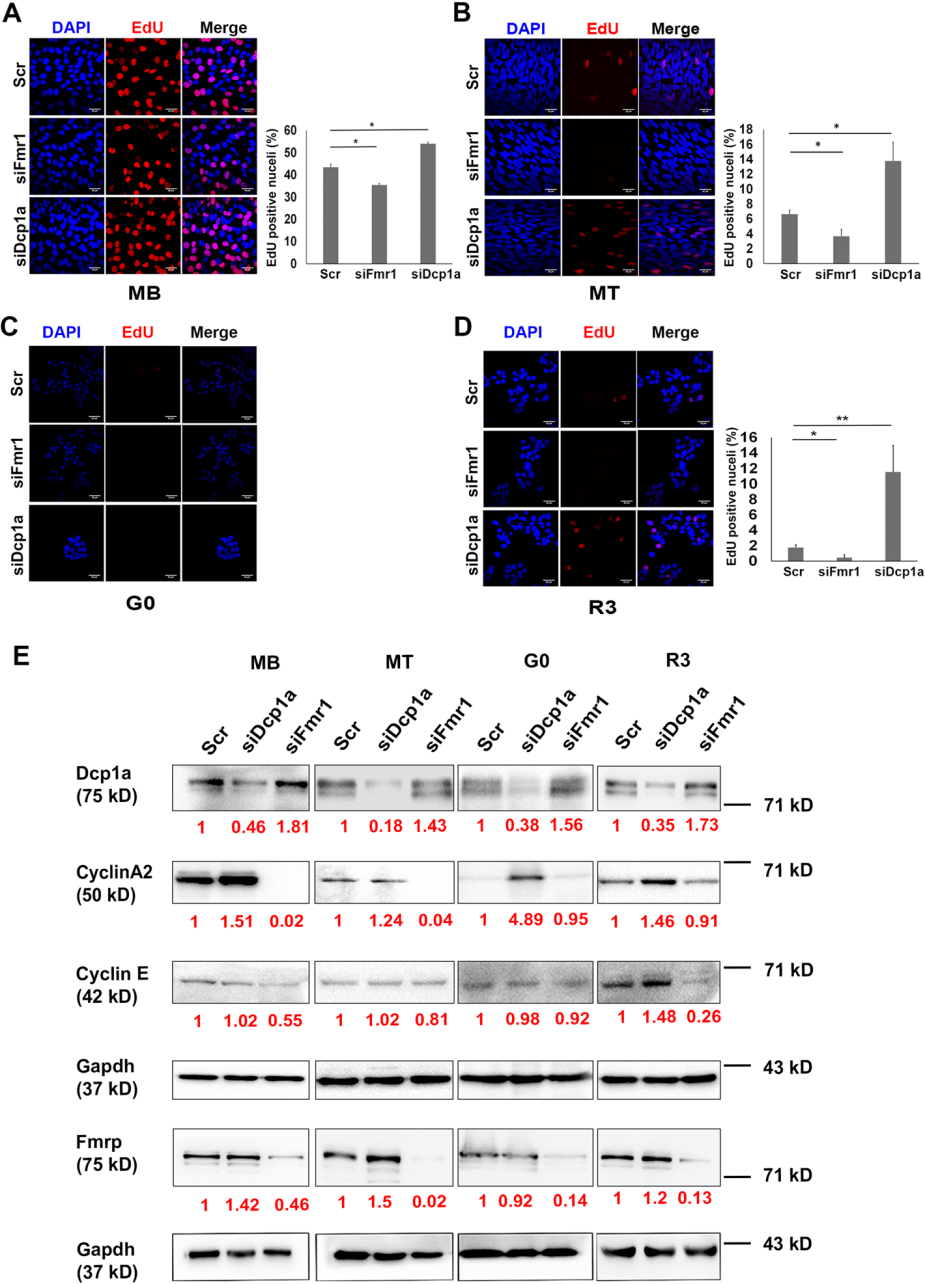
Knockdown of Fmrp and Dcp1a show opposing effects on the cell cycle. Proliferating myoblasts (MB) were treated with siRNAs (Scr, siDcp1a, siFmr1) for 18 h and either induced to enter G0 for 48 h or induced to differentiate for 48 h. For reactivation, G0 cells were harvested and plated on dishes or coverslips for 3 h. **a–d** EdU incorporation in MB (**a**), MT (**b**), G0 (**c**), and R3 (**d**): Dcp1a knockdown cells show increased incorporation of EdU in MB, MT, and R3, but not in G0, while Fmrp knockdown cells show decreased incorporation. EdU assay was performed simultaneously for all the conditions. Graphs show quantification by scoring > 500 nuclei for each condition in 3 biological replicates. * p < 0.05, ** p < 0.01. Two-tailed paired Student’s *t* test was performed. **e** Consistent with EdU incorporation, Cyclin A2 and Cyclin E show altered protein expression in Dcp1a and Fmrp knockdowns. Gapdh used as internal control. Values represent the mean + SD in 3 biological replicates

The results so far indicate differential mRNP granule protein abundance and assembly in distinct cellular states: the quiescent state is enriched in translational silencing/repressive complexes, whereas proliferating cells are enriched in the classical mRNA decapping and decay complexes. Further, reducing the abundance of translational repressor Fmrp by knockdown led to increased abundance and assembly of mRNA decay regulator Dcp1a and vice versa. To determine whether the differential enrichment of the decay and repressive complexes plays a role in the maintenance of a particular cellular state, we examined the phenotypes of the knockdown cells. Knockdown of Dcp1a in proliferating MB caused cells to proliferate more rapidly than control siRNA-treated cells, as evidenced by a significant increase in cell number and 5-Ethynyl-2´-deoxyuridine (EdU) incorporation (Fig. 8a). By contrast, knockdown of Fmrp led to reduced EdU incorporation (Fig. 8a), mimicking the reduced proliferative capacity seen in MuSC from *Fmr1* ^-/-^ mice (Fig. 3d). Together, these results indicate that compromising the expression of key decapping and repressive/silencing mRNP proteins differentially affects proliferation in myoblasts. Dcp1a and Fmrp thus exert opposing effects on cell proliferation, possibly by targeting different transcripts for degradation, translational repression, and/or sequestration. To identify the regulatory nodes at which Fmrp and Dcp1a might exert their effects, we evaluated the expression of cell cycle regulatory proteins by western blotting. In Dcp1a knockdown cells, Cyclin A2 protein expression increased, consistent with enhanced proliferation (Figs. 8e and S6A). In Fmrp knockdown cells, by contrast, Cyclin A2 and Cyclin E protein levels were decreased, consistent with reduced proliferative capacity (Figs. 8e and S6A).

### Knockdown of Fmrp and Dcp1a on cell cycle regulators during quiescence and reactivation

As knockdown of Fmrp and Dcp1a had opposing effects on MB proliferation, we evaluated the consequences of the knockdowns on expression of cell cycle regulators in G0 and R3. In G0 conditions, we found nearly 5-fold increase in Cyclin A2 protein expression when Dcp1a was knocked down (Figs. 8e and S6C), consistent with the increased proliferation observed in the MB condition. Fmrp knockdown, however did not affect protein expression of Cyclin A2 or Cyclin E (Figs. 8e and S6C), consistent with the unchanged EdU incorporation in either knockdown in G0 (Fig. 8c). Moreover, Fmrp knockdown G0 cells displayed reduced levels of mRNAs encoding *Cyclin A2, B1, E, D1*, and *ki67*, but negligible change in the levels of transcripts encoding either cell cycle inhibitors (C*dkn1a/p21, Cdkn1b/p27*) or myogenic regulatory factors (*MyoD*, *Pax7* and *Myf5*) (Fig. S7). By contrast, Dcp1a knockdown increased levels of pro-proliferative transcripts including *ki67* and *Cyclin A2, B1, E, and ki67,* and reduced levels of the anti-proliferative Cdk inhibitor, *Cdkn1a*/*p21*, consistent with increased proliferation. Interestingly, both *MyoD* and *Pax7* transcript levels were strongly reduced. Taken together, the reciprocal molecular phenotypes of Fmrp and Dcp1a knockdowns in cells in G0 were consistent with the observed reciprocal effect on proliferation.

Despite the altered mRNA profiles detailed above, both Dcp1a and Fmrp knockdown cells were able to undergo arrest as evidenced by the absence of DNA synthesis in suspension culture. However, whereas Dcp1a knockdown cells re-entered the cell cycle more rapidly (see below), Fmrp knockdown cells were deficient in reactivation. These results suggest that Fmrp may normally enable reversible quiescence. Therefore, we determined whether Fmrp knockdown cells activated other inhibitory programs, such as apoptosis or senescence. Flow cytometry of Fmrp knockdown cells revealed no increase in markers of apoptosis (Fig. S3B). Further, there was no increase in markers of senescence (Fig. S4). Yet, as referred to earlier, Fmrp knockdown cells showed poor colony-forming ability, indicating compromised self-renewal (Fig. S3A). Taken together, these results suggest that the absence of Fmrp leads to an altered G0 state that we term “aberrant quiescence”.

Indeed, knockdown of Dcp1a and Fmrp had a marked impact during reactivation of quiescent cells. At 3 h of reactivation, Dcp1a knockdown cells precociously displayed increased EdU incorporation compared with control cells (Fig. 8d). Supporting the premature entry into S phase, Dcp1a knockdown cells showed increased expression of both Cyclin A2 and Cyclin E proteins (Figs. 8e and S6D). By contrast, Fmrp knockdown cells showed even less EdU^**+**^ cells than the control at this early activation time point, and displayed strong suppression of Cyclin E protein levels (Figs. 8e and S6D), indicative of poor reactivation. Together, these data are consistent with opposing effects of Fmrp and Dcp1 on proliferation, and suggest that Fmrp and Dcp1a modulate quiescence entry/exit potentially by targeting stability/utilization of cyclin transcripts.

### Knockdown of either Fmrp or Dcp1a compromises myogenic differentiation

**Fig. 9:**
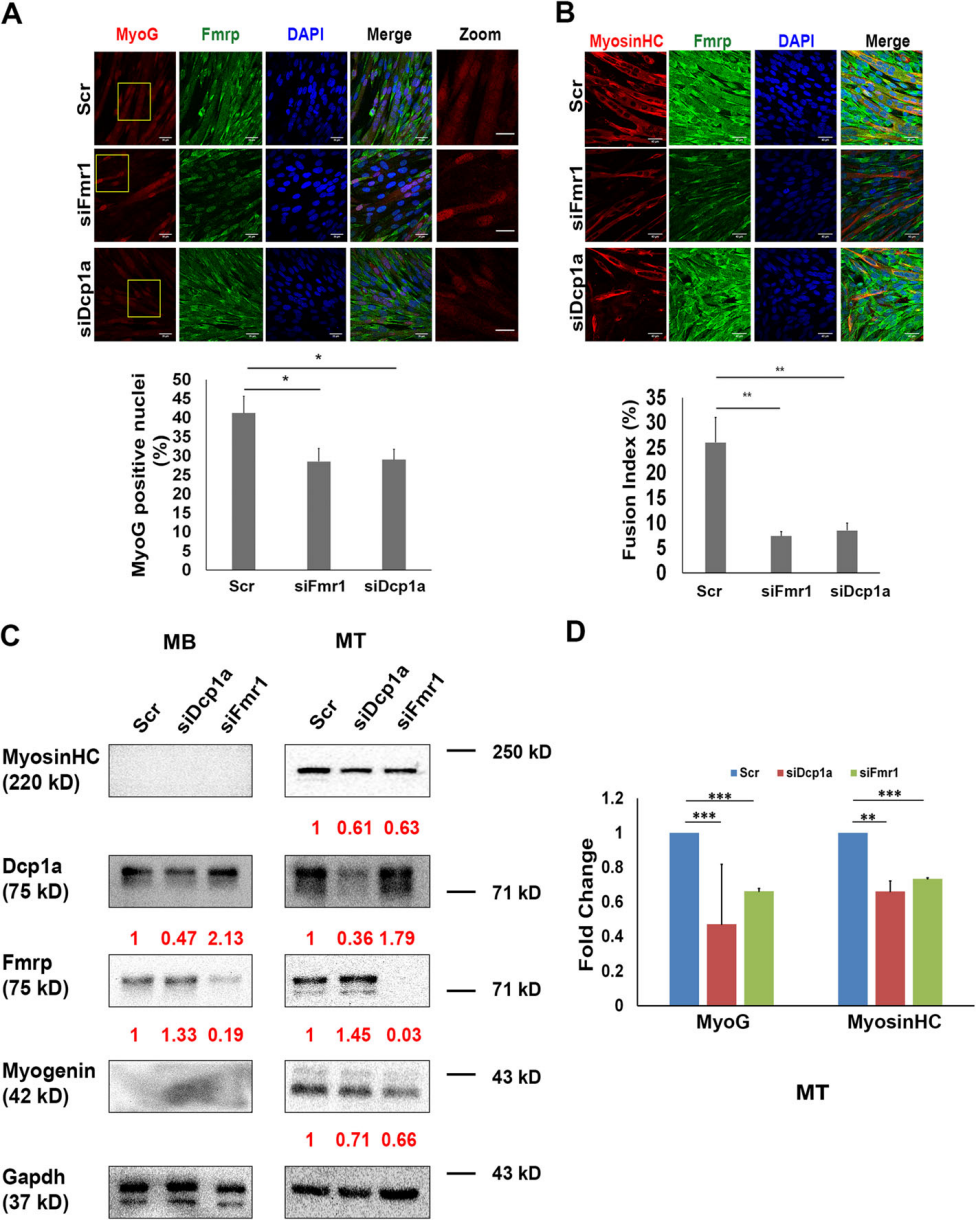
Knockdown of Fmrp and Dcp1a show similar effects on differentiation. Proliferating myoblasts (MB) were treated with siRNAs (Scr, siDcp1a, siFmr1) for 18 h and induced to differentiate for 48 h. **a** Both Fmr1 and Dcp1a knockdowns show reduced number of Myogenin^**+**^ nuclei. Upper Panel: Immunofluorescence of Myogenin (MyoG) and Fmrp in Scr, siFmr1 and siDcp1a. Scale bars represent 35 μm except in magnified panels where scale bars represent 17 μm. Lower panel: quantification based on 3 replicates, with > 600 nuclei scored per condition. **b** Knockdown of either Fmrp or Dcp1a affects fusion of myoblasts as shown by reduction in fusion index. Upper panel: immunofluorescence of myosin heavy chain (Myosin HC) and Fmrp in Scr, siFmr1, and siDcp1a. Lower panel: fusion index calculated as the ratio of the number of nuclei in myotubes with 2 or more nuclei over the total number of nuclei × 100 for *n* = 3 biological replicates. More than 850 nuclei were counted per condition. For **a** and **b** * *p* < 0.05, ** *p* < 0.01. Two-tailed Student’s *t* test was performed. **c** Representative western blots (from 3 biological replicates) of Myogenin (MyoG) and Myosin Heavy Chain (Myosin HC) proteins in MB and MT; Gapdh is internal control. **d** Densitometry of western blots of Myogenin (MyoG) and Myosin Heavy Chain (Myosin HC) proteins in MB and MT in **c**; Gapdh is loading control. Western blot analysis decreased expression of both myogenin and myosin when either Fmrp or Dcp1a expression is reduced. Two-tailed Student’s *t* test was performed, ** *p* < 0.01, *** *p* < 0.001. Values represent the mean + SD in 3 biological replicates

To assess the effects of depletion of Fmrp and Dcp1a on myogenesis, knockdown myoblasts were induced to differentiate for 2 days. As in proliferative conditions, Dcp1a knockdown in low serum conditions also led to sustained EdU incorporation with a corresponding increase in Cyclin A2 protein (Fig, 8b, e and S6B). By contrast, Fmrp knockdown lead to negligible EdU incorporation accompanied by drastic reduction in Cyclin A2 protein compared with control. Notably, the cross-regulation of Fmrp by Dcp1a knockdown (*see previous section*) was most pronounced in myotubes, and correlated with a pronounced suppression of Myogenin protein in the same sample, consistent with translation suppressive function of Fmrp. However, maintenance of the knockdown cells in differentiation conditions showed that loss of either Fmrp and Dcp1a negatively affected differentiation as evidenced by reduced Myogenin protein abundance, decreased frequency of Myogenin^**+**^ nuclei, reduced Myosin Heavy Chain protein expression and significantly reduced fusion index (Figs. 9a–d and S6E). Taken together, these results indicate that despite their opposing effects on the cell cycle, optimal levels of both Dcp1a and Fmrp are required for myogenesis.

**Fig. 10:**
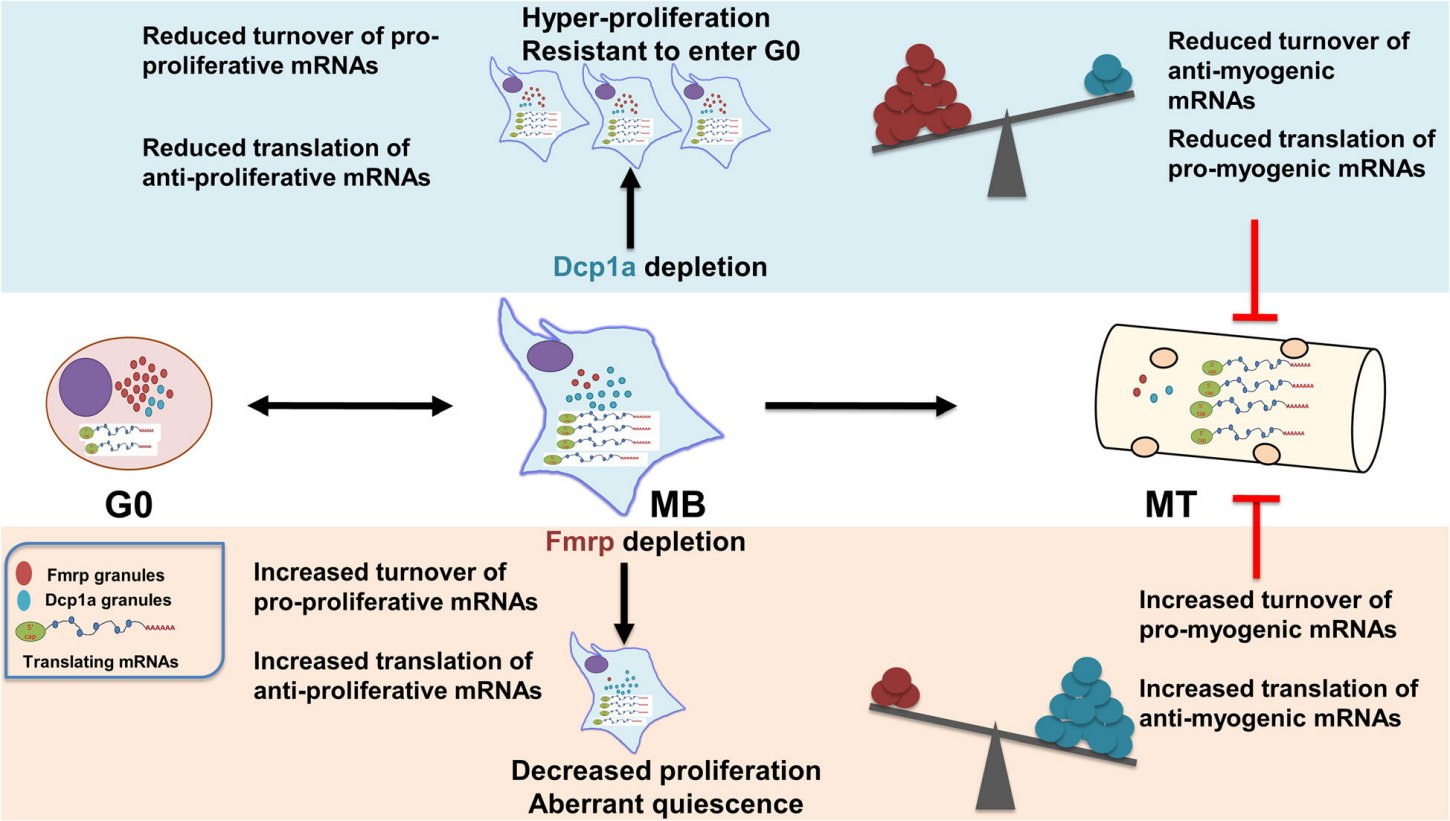
Model showing how cross-regulation of Dcp1a and Fmrp alters the balance of mRNA turnover and translation. The balance of Dcp1a and Fmrp are hypothesized to control the turnover and translation of different sets of transcripts in distinct cellular states (middle row). When wild-type proliferating myoblasts (MB, center) enter quiescence (G0, left), protein synthesis is repression and stalled polysomes are detected, paralleled by enrichment of the translational repressor Fmrp into prominent puncta, whereas Dcp1a puncta diminish. In contrast, differentiation (MT, right) is associated with a reduction of both Fmrp and Dcp1a puncta, suggesting a new set point for the balance of these regulators. Perturbing expression of Dcp1a (upper row) or Fmrp (lower row) has reciprocal effects on mRNP granules, and opposing phenotypic consequences. Depletion of Dcp1a leads to increased Fmrp accumulation and assembly, whereas depletion of Fmrp leads to increased Dcp1a accumulation and assembly. Dcp1a knockdown (upper row) may increase levels of proteins that enhance cell proliferation directly (via reduced mRNA turnover), and indirectly act via increasing Fmrp to reduce translation of negative cell cycle regulators. Such hyper-proliferative Dcp1a knockdown cells are resistant to induction of quiescence. Conversely, Fmrp knockdown (lower row) may increase levels of proteins that repress the cell cycle directly (via de-repressed translation), and indirectly decrease levels of proteins that positively regulate the cell cycle (via increased Dcp1a and increased turnover of transcripts). Thus, Fmrp knockdown cells show reduced cell proliferation and Dcp1a knockdown cells show increased proliferation. The observations that normal induction of quiescence leads to increased Fmrp accumulation, whereas forced suppression of Fmrp also decreases proliferation, suggest that a threshold of Fmrp accumulation/assembly is required to balance between proliferation and quiescence. The observation that depletion of either Dcp1a or Fmrp leads to compromised differentiation may be explained by altered net translation of different sets of pro- and anti-myogenic target transcripts

In summary, our data support a model (Fig. 10), where Fmrp and Dcp1a reciprocally regulate each other at the level of protein abundance and granule assembly, differentially regulate the expression of cell cycle and myogenic proteins and thereby play critical and opposing roles in the transitions between proliferation and reversible quiescence, ultimately leading to compromised differentiation.

## Discussion

In this study, we show that components of mRNP granules regulate MuSC proliferation and differentiation in vitro and myogenesis in vivo, likely through changes in the translation and turnover of mRNAs encoding key regulators of MuSC dynamics.

### Quiescent cells display distinct mRNP complexes

Non-dividing cells are well-known to exhibit reduced macromolecular metabolism. Here, we show that muscle cells in two distinct states of cell cycle arrest elaborate distinct mRNP granule protein expression, correlating with global protein synthesis. When MB enter permanent arrest associated with differentiation to MT, robust levels of protein synthesis sustain tissue-specific functions. However, in reversible arrest (G0), which is typical of adult stem cells, protein synthesis is restricted, and cells enter a suppressed state that is poised for reactivation. Strikingly, proteins involved in mRNA degradation are enriched in MB, while G0 cells are enriched in proteins involved in mRNA storage/suppression of translation. In particular, quiescence is characterized by reduced expression of initiation factors, low rates of protein synthesis, and potentially, stalled polysomes.

Assembly of mRNP components into granules also differs between G0 and MT. mRNP granules are assembled around distinct transcripts and modulate their functionality. These mRNP-associated transcripts may either be degraded, or remain in a stable, untranslated state, where the composition of a particular mRNP complex determines the fate of individual transcripts. Our study reveals that in culture, mRNP granules containing decapping proteins of the classical decay complex (Dcp1a, Pat1, Edc4) (Table 3) are enriched in MB, suggesting that ‘stockpiling’ of inactive transcripts in quiescence as during embryogenesis [50], may facilitate cell cycle reentry when translation resumes. Notably, during G0, translationally repressive complexes (Fmrp+) dominate, consistent with the enrichment of Fmrp+ storage granules in quiescent muscle stem cells in vivo [17] (Fig. 1). Our findings confirm the recent report [23] that Fmrp is required for MuSC function in vivo.

**Table 3:** Details of antibodies used in this study

Antibody	Species	Company	Catalog #	Dilution for WB	Dilution for IFA
Argonaute2	Rabbit	CST	C34C6	1 in 1000	1 in 200
Cyclin A2	Rabbit	Abcam	ab181591	1 in 2000	
Cyclin E	Rabbit	Abcam	ab71535	1 in 1000	
Dcp1a	Mouse	Santa Cruz	sc100706	1 in 200	1 in 50
Edc3	Mouse	Santa Cruz	sc365024	1 in 200	1 in 50
Edc4	Rabbit	Santa Cruz	ab72408	1 in 200	1 in 50
eIF4E	Mouse	Santa Cruz	sc271480	1 in 200	1 in 50
Eif4g	Goat	Santa Cruz	sc9602	1 in 200	1 in 50
Fmrp	Rabbit	Sigma	4055	1 in 1000	1 in 200
FxR1	Goat	Abcam	ab51970	1 in 1000	
Gapdh	Mouse	Abcam	ab8245	1 in 10000	
GFP	Chicken	Abcam	ab13970		1 in 300
GW182	Mouse	Santa Cruz	sc56314	1 in 200	1 in 50
γH2AX (Ser139)	Mouse	Upstate	05636		1 in 200
MyoD	Mouse	Dako	M3512	1 in 250	1 in 200
MyoG	Mouse	Santa Cruz	sc12732	1 in 250	1 in 250
Myosin Heavy Chain	Mouse	Hybridoma	A4.1025	1 in 1	1 in 1
Pabp1	Rabbit	Abcam	ab21060		1 in 50
Pax7	Mouse	Aviva	ARP30947_P050	1in 100	
RLP0	Rabbit	Abcam	ab101279	1 in 1000	
Tia1	Mouse	Santa Cruz	sc166247	1 in 200	1 in 50
TiaR	Goat	Santa Cruz	sc1749	1 in 200	1 in 50
Xrn1	Rabbit	Sigma	sab4200028	1 in 1000	1 in 200

mRNP puncta are thought to represent sites where the mRNP granule proteins exert their function [41, 51]. The increased abundance of Fmrp puncta in G0 may suggest a role either in the entry into or maintenance of quiescence. By contrast, the reduced Dcp1a puncta would suggest that Dcp1a either opposes or is not important for quiescence. As discussed in detail below, the functional data is in apparent contradiction with this interpretation: knocking down Fmrp expression (leading to lower Fmrp puncta accumulation) slows the cell cycle, whereas knocking down Dcp1a hastens the cell cycle. When Fmrp expression is compromised, the cells enter into an aberrant quiescence, from which they are unable to exit. The aberrant quiescent state is not accompanied by increased cell death or activation of senescence markers, but does exhibit compromised clonogenicity (self-renewal), and warrants further investigation. A possible explanation is that Fmrp plays a role in the translational pausing we observed in the primed or poised quiescent state, as first reported by Crist *et.al.,* [17]. If in absence of Fmrp its target mRNAs are continuously translated, the cell might be unable to leave quiescence. Another possibility is that, as Dcp1a expression and assembly are enhanced in Fmrp knockdown cells, transcripts that would normally be stabilized in a translationally repressed state (associated with Fmrp) now become targets for more rapid turnover by Dcp1a. We hypothesize that among these destabilized transcripts would be those required for the exit from quiescence. A detailed understanding of the direct and indirect targets of Fmrp and Dcp1a in different cellular states is needed to resolve this issue.

## Reciprocal effects of Fmrp and Dcp1a on the cell cycle may reflect the balance between mRNA turnover and translation in control of cell state

Perturbing Fmrp and Dcp1a expression in proliferating cells had contrasting impacts on the cell cycle. In cycling cells, Fmrp knockdown led to an increase in Dcp1a and a simultaneous reduction in EdU incorporation, suggesting that increased nonsense-mediated decay may lead to degradation of target mRNAs, and compromise S phase entry. Support for this hypothesis comes from the observation that expression of *Cyclin E*, a key positive regulator of the G1/S transition is suppressed in the Fmrp knockdown. Given the enrichment of Fmrp stalling complexes and the severe translational block in G0, a requirement for Fmrp in sustaining expression of *Cyclin E* may appear paradoxical. However, it is also possible that diminished expression of *Cyclin E* reflects increased Dcp1a protein abundance and increased formation of Dcp1a puncta in Fmrp knockdown cells.

By contrast, Dcp1a knockdown enhanced S phase entry, and enhanced mRNA levels of positive regulators of progression – *Cyclins A2, B2,* and *D1*. These changes may be a direct effect of reduced cyclin mRNA turnover. However, given the concomitant increase in Fmrp expression and puncta, indirect effects of Dcp1a knockdown on negative regulators of the cell cycle cannot be ruled out. For example, reduced translation of a potential Fmrp target such as *Cdkn1a/p21* would synergize with increased cyclin mRNA expression to enhance S phase entry. The identity of direct and indirect targets of Fmrp and Dcp1a in different cellular states are currently not known, and would likely resolve this conundrum.

The opposing phenotypes of the Dcp1a and Fmrp knockdown in MB are consistent with the opposing roles played by these proteins in regulation of the cell cycle. These phenotypic changes are sustained in both quiescent as well as reactivated conditions. Specifically, increased pro-proliferative transcripts and decreased cell cycle inhibitor transcripts are observed in Dcp1a knockdown cells in G0, and the converse in Fmrp knockdown cells. However, the increased Cyclin A2 protein expression in Dcp1a knockdown is not accompanied by increased EdU incorporation in G0, suggesting that other elements act to maintain quiescence.

The effects of Fmrp knockdown on the activation out of quiescence in culture were reminiscent of the restricted proliferation of *Fmr1*^*-/-*^ MuSCs, reflected by unchanged EdU incorporation and decreased Cyclin A2 and Cyclin E. Fmrp may directly target cell cycle transcripts, blocking their translation in quiescence, but stabilizing them for mobilization during reactivation. In absence of Fmrp during cell cycle entry, these transcripts may instead be targeted for translation and turnover, by the increase in Dcp1a. The phenotype of Dcp1a knockdown during cell cycle re-entry from G0 was similar to that of cycling myoblasts: increased EdU incorporation accompanied by increased Cyclin A2 and Cyclin E expression. As mRNP puncta are assembled in cell cycle activated myoblasts within 3 h, the Dcp1a knockdown may accelerate proliferation via increased accumulation of pro-cell cycle transcripts and increased translation, rather than sequestration.

All cellular states express some transcripts with extremely short half-lives, and likely targets of Dcp1a. The work of the Coller group [52] has shown that genome-wide half-lives of transcripts are increased during quiescence in human fibroblasts, but regulators of this change were not identified. The targets of Dcp1a in different cellular states are likely to be numerous and context-specific, as suggested by studies in oocytes and embryos (Ma et al (2013); Eulalio et al, (2007) [53, 54]. In our study, we show that transcripts of cyclins A2, B1, D1, E and Ki67 are increased when Dcp1a expression is compromised in G0, providing possible direct targets. The model in Fig. 10 outlines the possible targets in other states, suggesting reduced turnover and translation of pro-proliferative transcripts during quiescence and pro-myogenic differentiation respectively in Dcp1a-depleted cells. By contrast, non-translating mRNAs held in repressive mRNP granule complexes containing Fmrp would be expected to become targets for increased turnover under conditions where Dcp1a is induced by knockdown of Fmrp. Considering the compromised proliferation of Fmrp knockdown cells, we hypothesize that pro-proliferative transcripts are likely targets for rapid turnover, as depicted in the model in Fig. 10. Indeed, our finding that mRNAs for Cyclins D, E A, and B all show decreased levels in the Fmrp knockdown and increased levels in the Dcp1a knockdown (Fig. S7) is consistent with this scenario.

### Both Fmrp and Dcp1a are necessary for normal differentiation

Whereas Fmrp and Dcp1a have opposing effects on proliferation consistent with their opposing functions in mRNA turnover vs. translation, differentiation is suppressed when either Fmrp or Dcp1a are perturbed. Although the direct targets are currently unknown, the mechanisms by which these two regulators affect myogenesis are likely to differ. As Fmrp knockdown leads to reduced proliferative capacity, reduced differentiation may reflect the reduced number of cells available for myogenic commitment. It has been reported [23] that *Fmr1*^*-/-*^ MuSCs show lower accumulation of MyoD and Myf5 proteins through translational silencing, delaying entry into the differentiation program. Our study suggests that this effect could be at multiple regulatory nodes where Fmrp either directly or indirectly participates in decisions regarding cell fate. The effect of Dcp1a knockdown on differentiation is consistent with the observed increase in proliferation, the antagonistic nature of these programs being well reported. At a mechanistic level, the loss of differentiation potential may reflect the strong reduction of Myogenin protein.

### Cross regulation of mRNP granule components revealed by knockdown analysis

Knockdown of Fmrp resulted in an increase in Dcp1a puncta, and knockdown of Dcp1a led to an increase of Fmrp in puncta (Fig. 7b) suggesting a reciprocal balance between mRNA decay and translational repression. Specifically, our results point to a regulatory loop where Fmrp negatively regulates Dcp1a function and Dcp1a negatively regulates Fmrp function (Fig. 10). Our observations may be explained by a model wherein the translation repression normally effected by Fmrp on target mRNAs in proliferating myoblasts would be lifted in the Fmrp knockdown, with consequent increase in Dcp1a-associated NMD complex resulting in possible degradation of transcripts including cyclins. The knockdown of Dcp1a could also lead to a decrease in the ARE-mediated decay pathway [54, 55] leading to increased half-life of cyclin and cytokine transcripts, potentiating cell cycle progression by preventing entry into G0 [26, 28]. Conceivably, altering the flux of different transcripts through distinct puncta could alter the profile of proteins synthesized, impacting proliferation.

The molecular mechanism for reciprocal regulation that we observe in Fmrp and Dcp1a knockdown muscle cells may involve direct mRNA binding by each protein, or may be mediated by indirect regulation of upstream regulators. However, as knockdown of Fmrp and Dcp1a each have broad phenotypic consequences for the cell cycle and differentiation, it is also possible that the altered levels of each protein (in the context of knockdown of the other) are associated with the altered cellular state. At present, we cannot distinguish whether the mechanisms involving mRNP granules we describe are directly responsible for regulating MuSC fate, or a consequence of signaling that affects global translation and that consequently impacts mRNP granules.

Overall, these observations indicate that both Dcp1a and Fmrp may play a role in the assembly of mRNP complexes, and that individually their knockdown affects the expression of transcripts encoding other mRNP proteins. Dcp1a knockdown had more pronounced effects on transcript abundance than Fmrp knockdown, consistent with the expected differential effects of mRNA degradation versus translational stalling. Thus, Dcp1a knockdown, by altering transcript levels of Fmr1 a key mRNP player in G0-inducing conditions, may alter the equilibrium between mRNA decay and sequestration required for achieving and maintaining the quiescent state. Our studies point to integrative mechanisms regulating a critical balance between the mRNA decay and translational repression, which enables expression of cell cycle (and other) regulators that control proliferation, quiescence or differentiation. In summary, our results support a model where distinct mRNP constellations characterize different cellular states and suggest that remodeling these complexes may contribute to the transitions between states.

## Supplementary Information

**Additional file 1: Table 1**. Bio-informatic analysis of transcripts encoding mRNP components. To assess whether changes in expression of mRNP proteins resulted from changes in expression of their mRNAs, we used the recent RNA seq analysis derived from muscle satellite cells fixed by perfusion of adult mice (to prevent cell activation that results from disruption of the niche during isolation [42]. These fixed satellite cells are thought to more accurately represent the quiescent (G0 state) and have a transcriptome profile distinct from MuSC isolated without fixation, which are now understood to represent cells in an early activation state. Activated satellite cells (ASC) represent proliferating primary myoblasts 2.5 days post isolation from the animal. Transcripts encoding P body genes were selected from the RNAseq data and grouped according to their function as outlined [43, 44]. We calculated fold changes from FPKM values (Fragments Per Kilobase of transcript per Million mapped reads) RNA seq data comparing fixed (quiescent) satellite cells and activated satellite cells [44] and used a cut-off of 1.5 +/- (for up regulation and down regulation). False Discovery Rate approach: Two stage step-up method of Benjamini, Krieger and Yekutieli was used and 10% FDR was set up for generating p values for the analysis. **Figure S1**. *Differential association of decay complex proteins in different cellular states.* Immuno-staining of Dcp1a/Edc4/Pat1 (left) and Dcp1a/Ago2 (right) in muscle cells in culture: quiescent (G0), 3 hr reactivated (R3), proliferative (MB), and differentiated (MT). Blue arrows indicate co-localization of Dcp1a/Edc4/Pat1 in puncta. Red arrows indicate co-localization of Dcp1a/Ago2 in puncta. Note the absence of Dcp1a or Pat1 puncta in G0, and the rapid reassembly in R3. Also note prominent nuclear staining for Edc4 in G0. **Figure S2**. (A) Supplementary to Figure 4A Additional representative immunofluorescence images showing Fmrp (green) and Dcp1a (red) puncta in G0, MB and MT, as well as cells reactivated for 3 hr from G0 (R3). Arrows indicate prominent puncta. (B). Corrected Mean Fluorescence intensities (CMI) of Fmrp and Dcp1a respectively in MB, G0, R3 and MT. For quantification, more than 3 cells per group was used and CMI intensities from more than 12 puncta were analysed. Corrected mean intensity was calculated using CMI= Total intensity of signal – (Area of signal x Mean background signal). For quantification, more than 3 cells per group was used and MFI intensities from more than 10 puncta was analysed. **Figure S3**. *Knockdown of Fmrp leads to reduced cell renewability and is not accompanied by apoptosis.* (A) Colony formation assay shows that reduced EdU incorporation in Fmrp knockdown cells correlates with compromised self-renewal. Bar graph represents mean ± sd from n=3 biological replicates. Two tailed paired Student’s t-test is indicated as ***p<0.001. (B) Proliferating myoblasts (MB) were treated with siRNAs (Scr, siFmr1) for 18 hr and harvested at 24 hrs for FACS analysis of 10,000 cells performed after staining for apoptosis markers. X-axis represents Annexin V and Y-axis represents propidium iodide. Upper Panel: Flow cytometric profile for MB. Lower Panel: Quantification of FACS plots shows that Fmrp knockdown cells do not undergo apoptosis. *n*=3, mean ± sd. **Figure S4**. *Knockdown of Fmrp is not accompanied by senescence.* (A). SA β-galactosidase assay performed in MB cells treated with siRNAs (Scr, siFmr1) for 24 h or reactivated from quiescence for proliferation for 24 hrs (R24) does not show any significant difference in X-gal staining between control and Fmr1 Knock down cells. (B). Analysis of DNA damage-induced foci of γH2AX in cells reactivated from quiescence for 24h (R24) does not reveal any increase in Fmr1 knock down cells. (C). qRT-PCR analysis for p21 did not reveal any significant change in Fmr1 knocked down condition in MB, G0 or R24. All bar graphs represent mean ± sd from n =2. Two tailed paired Student’s t-test is indicated as ***p <0.001. **Figure S5**. (A): *Phenotyping of 3 cellular conditions*: A replicate blot is shown of the data depicted in Figure 4C. Myogenin is exclusively expressed by MT, Cyclin D1 is enriched in MB, and G0 cells lack both proteins, confirming their quiescence by the absence of both proliferation and differentiation programs. (B) *Global Transcript status*: The global transcript levels of Cyclin D1, p27, MyoD1, Myf5, Gapdh in MB, G0, MT were analysed using data sets from Venugopal *et.al.,* [49] 2020. GEO database (Series GSE110742). *p < 0.05. **p < 0.01, ***p <0.001. **Figure S6**. *Dcp1a and Fmrp Knockdowns have opposing effects on cell proliferation but compromise differentiation.* This data represents quantification of western blots shown in Figure 8. (A to D): Densitometry of western blots of Dcp1a, Fmrp , Cyclin A2 , Cyclin E proteins normalized with Gapdh as internal control in MB , MT, G0 and R3 (E): Western blots of Myogenin and Myosin Heavy chain proteins in G0 and R3 with Gapdh as internal control. All bar graphs represent mean ± sd from n ≥ 3 ,Two tailed paired Student’s t-test is indicated is indicated as *p < 0.05. *p < 0.01, ***p <0.001. **Figure S7**. *Altered expression of cell cycle and myogenic transcripts in knockdown cells held in G0-inducing conditions.* Cells were transfected with siFmr1 and siDcp1a pools for 18 hours, then placed in suspension culture and 48 hours later RNA was isolated for qRT-PCR of Dcp1a, Fmr1, Ki67, Cyclin A2, Cyclin D1, Cyclin E1, Cyclin B, p27, p21, MyoD1, and Myf 5 Loss of Dcp1a leads to increased abundance of transcripts encoding positive regulators of the cell cycle (Ki67, Cyclins), along with suppression of Cdk inhibitor p21 mRNA levels, consistent with increased EdU incorporation. Transcripts encoding Pax7 and MyoD were suppressed. Knockdown of Fmr1 leads to reduced abundance of Cyclin A2, B and D1 mRNAs, consistent with decreased EdU incorporation. Gapdh was used as internal control and normalized to Scr G0 condition. All bar graphs represent mean ± sd from n =3. Two tailed paired Student’s t-test is indicated is indicated as *p < 0.05. **p < 0.01, ***p <0.001.

## Data Availability

All mouse strains, cell lines, antibodies, and siRNAs are available commercially.

## Abbreviations

Ago2: Argonaute 2
CD45: Cluster of Differentiation 45
Dcp1a: Decapping Protein 1a
Edc3: Enhancer of Decapping 3
Edc4: Enhancer of Decapping 4
EdU: 5-Ethynyl-2´-deoxyuridine
eIF-4E: Eukaryotic initiation factor 4E
eIF-4F: Eukaryotic initiation factor 4F
Fmrp: Fragile X Mental Retardation Protein
Ki67: Marker of Proliferation
LSm4: U6 snRNA-associated Sm-like protein LSm4
mRNP: Messenger Ribonucleoprotein
MuSC: Muscle Stem Cells
Myf5: Myogenic factor 5
MyoD: Myogenic Determination Protein 1
OPP: O-Propargyl Puromycin
P0: 60S acidic ribosomal protein P0
Pat1: DNA topoisomerase 2-associated protein PAT1
Pax7: Paired box 7
PFA: Para-formaldehyde
PB: Processing bodies
Scr: Scrambled
SG: Stress granules
TA: Tibialis anterior
TIA1: Tia1 cytotoxic granule-associated RNA binding protein
VCAM-1: Vascular cell adhesion molecule 1
