# Supplementary figures and images for: mRNP granule proteins Fmrp and Dcp1a differentially regulate mRNP complexes to contribute to control of muscle stem cell quiescence and activation
Source: Skelet Muscle. 2021 Jul 8;11:18. doi: 10.1186/s13395-021-00270-9 (PMC8265057; doi:10.1186/s13395-021-00270-9)

Figure S7

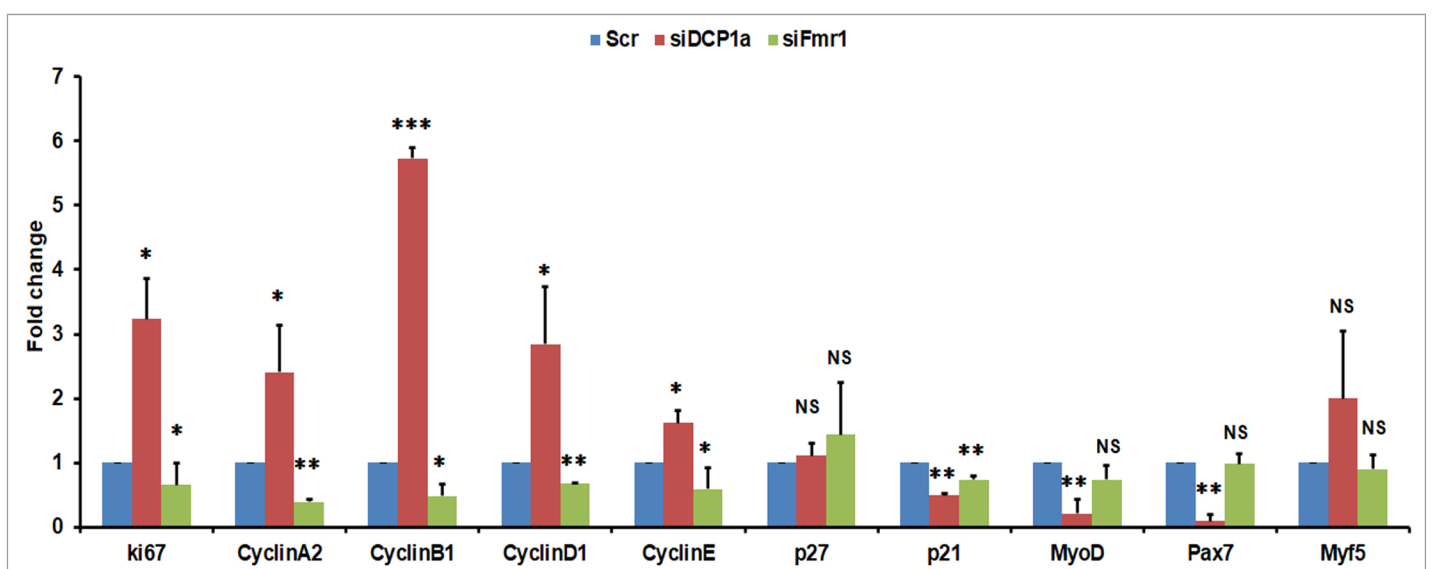

Supplement: Supplementary file 1 — Additional file 1: Table 1. Bio-informatic analysis of transcripts encoding mRNP components. To assess whether changes in expression of mRNP proteins resulted from changes in expression of their mRNAs, we used the recent RNA seq analysis derived from muscle satellite cells fixed by perfusion of adult mice (to prevent cell activation that results from disruption of the niche during isolation [42]. These fixed satellite cells are thought to more accurately represent the quiescent (G0 state) and have a transcriptome profile distinct from MuSC isolated without fixation, which are now understood to represent cells in an early activation state. Activated satellite cells (ASC) represent proliferating primary myoblasts 2.5 days post isolation from the animal. Transcripts encoding P body genes were selected from the RNAseq data and grouped according to their function as outlined [43, 44]. We calculated fold changes from FPKM values (Fragments Per Kilobase of transcript per Million mapped reads) RNA seq data comparing fixed (quiescent) satellite cells and activated satellite cells [44] and used a cut-off of 1.5 +/- (for up regulation and down regulation). False Discovery Rate approach: Two stage step-up method of Benjamini, Krieger and Yekutieli was used and 10% FDR was set up for generating p values for the analysis. Figure S1. Differential association of decay complex proteins in different cellular states. Immuno-staining of Dcp1a/Edc4/Pat1 (left) and Dcp1a/Ago2 (right) in muscle cells in culture: quiescent (G0), 3 hr reactivated (R3), proliferative (MB), and differentiated (MT). Blue arrows indicate co-localization of Dcp1a/Edc4/Pat1 in puncta. Red arrows indicate co-localization of Dcp1a/Ago2 in puncta. Note the absence of Dcp1a or Pat1 puncta in G0, and the rapid reassembly in R3. Also note prominent nuclear staining for Edc4 in G0. Figure S2. (A) Supplementary to Figure 4A Additional representative immunofluorescence images showing Fmrp (green) and Dcp1a (red) puncta in [file 13395_2021_270_MOESM1_ESM.zip › Roy et al rev2 Fig S7_ESM.pdf]

**Figure S6**

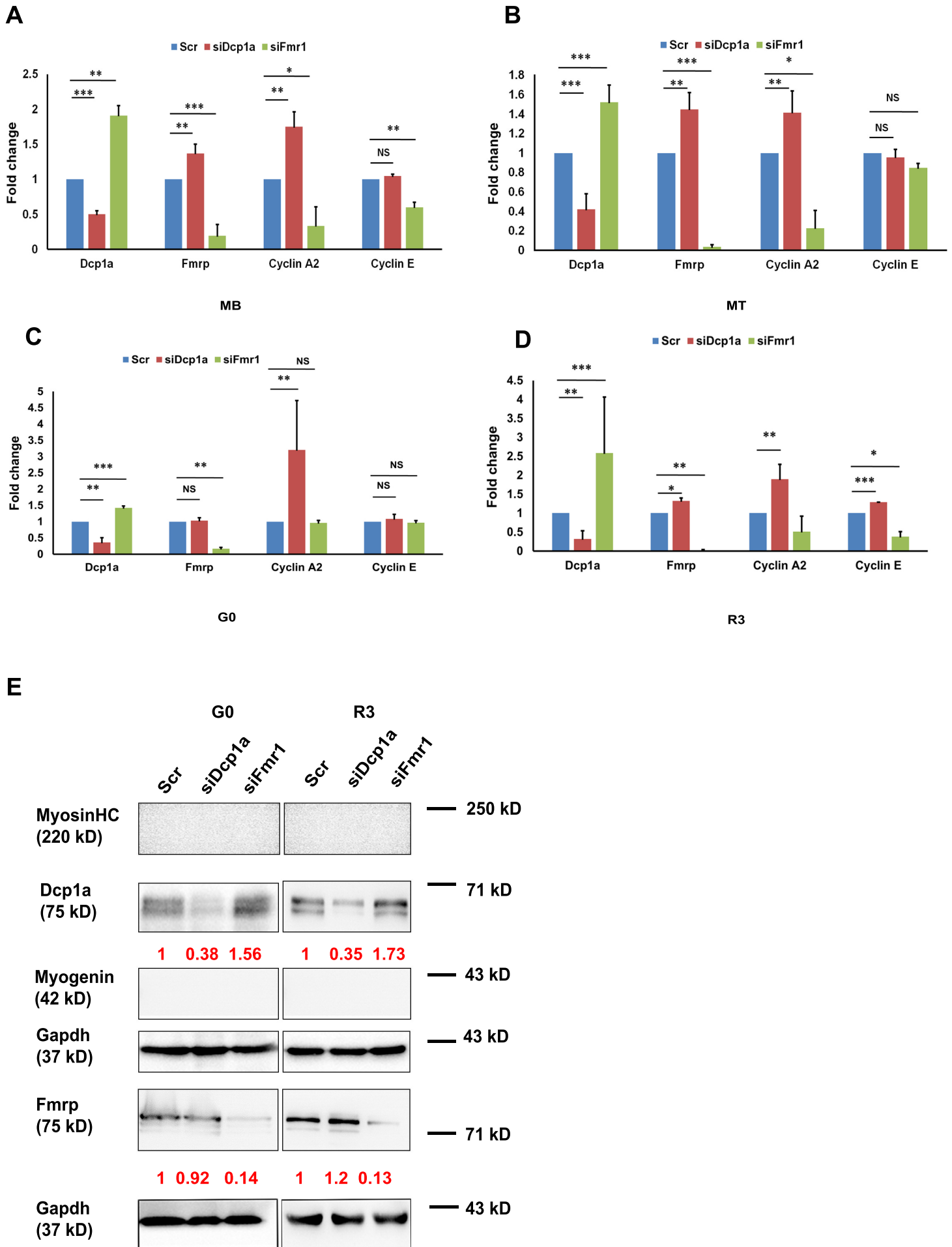

Supplement: Supplementary file 1 — Additional file 1: Table 1. Bio-informatic analysis of transcripts encoding mRNP components. To assess whether changes in expression of mRNP proteins resulted from changes in expression of their mRNAs, we used the recent RNA seq analysis derived from muscle satellite cells fixed by perfusion of adult mice (to prevent cell activation that results from disruption of the niche during isolation [42]. These fixed satellite cells are thought to more accurately represent the quiescent (G0 state) and have a transcriptome profile distinct from MuSC isolated without fixation, which are now understood to represent cells in an early activation state. Activated satellite cells (ASC) represent proliferating primary myoblasts 2.5 days post isolation from the animal. Transcripts encoding P body genes were selected from the RNAseq data and grouped according to their function as outlined [43, 44]. We calculated fold changes from FPKM values (Fragments Per Kilobase of transcript per Million mapped reads) RNA seq data comparing fixed (quiescent) satellite cells and activated satellite cells [44] and used a cut-off of 1.5 +/- (for up regulation and down regulation). False Discovery Rate approach: Two stage step-up method of Benjamini, Krieger and Yekutieli was used and 10% FDR was set up for generating p values for the analysis. Figure S1. Differential association of decay complex proteins in different cellular states. Immuno-staining of Dcp1a/Edc4/Pat1 (left) and Dcp1a/Ago2 (right) in muscle cells in culture: quiescent (G0), 3 hr reactivated (R3), proliferative (MB), and differentiated (MT). Blue arrows indicate co-localization of Dcp1a/Edc4/Pat1 in puncta. Red arrows indicate co-localization of Dcp1a/Ago2 in puncta. Note the absence of Dcp1a or Pat1 puncta in G0, and the rapid reassembly in R3. Also note prominent nuclear staining for Edc4 in G0. Figure S2. (A) Supplementary to Figure 4A Additional representative immunofluorescence images showing Fmrp (green) and Dcp1a (red) puncta in [file 13395_2021_270_MOESM1_ESM.zip › Roy et al rev2 Fig S6_ESM.pdf]

A

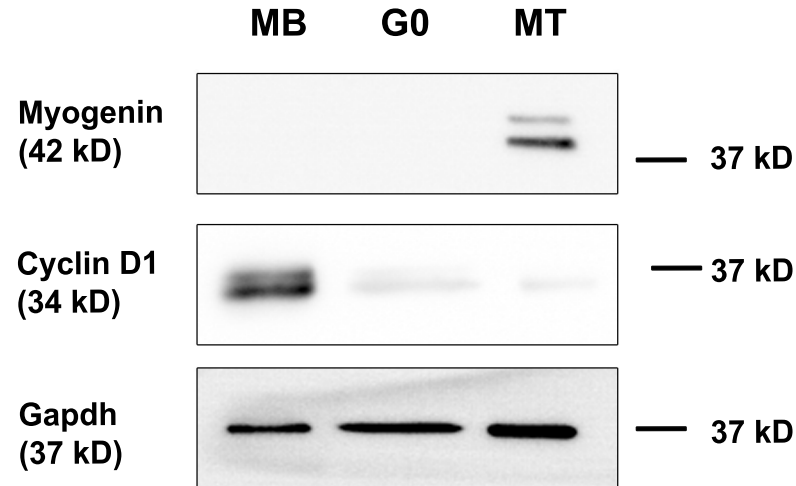

B

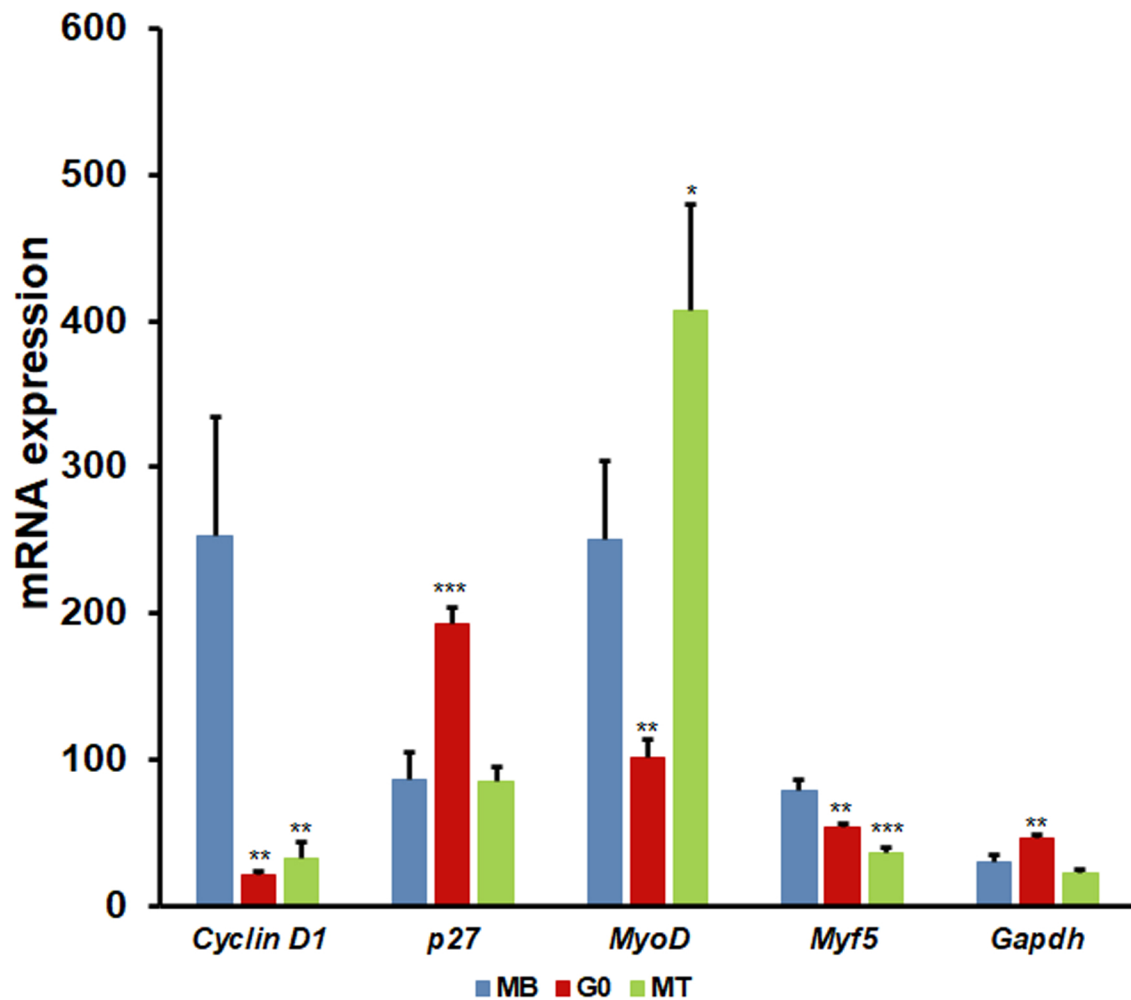

Supplement: Supplementary file 1 — Additional file 1: Table 1. Bio-informatic analysis of transcripts encoding mRNP components. To assess whether changes in expression of mRNP proteins resulted from changes in expression of their mRNAs, we used the recent RNA seq analysis derived from muscle satellite cells fixed by perfusion of adult mice (to prevent cell activation that results from disruption of the niche during isolation [42]. These fixed satellite cells are thought to more accurately represent the quiescent (G0 state) and have a transcriptome profile distinct from MuSC isolated without fixation, which are now understood to represent cells in an early activation state. Activated satellite cells (ASC) represent proliferating primary myoblasts 2.5 days post isolation from the animal. Transcripts encoding P body genes were selected from the RNAseq data and grouped according to their function as outlined [43, 44]. We calculated fold changes from FPKM values (Fragments Per Kilobase of transcript per Million mapped reads) RNA seq data comparing fixed (quiescent) satellite cells and activated satellite cells [44] and used a cut-off of 1.5 +/- (for up regulation and down regulation). False Discovery Rate approach: Two stage step-up method of Benjamini, Krieger and Yekutieli was used and 10% FDR was set up for generating p values for the analysis. Figure S1. Differential association of decay complex proteins in different cellular states. Immuno-staining of Dcp1a/Edc4/Pat1 (left) and Dcp1a/Ago2 (right) in muscle cells in culture: quiescent (G0), 3 hr reactivated (R3), proliferative (MB), and differentiated (MT). Blue arrows indicate co-localization of Dcp1a/Edc4/Pat1 in puncta. Red arrows indicate co-localization of Dcp1a/Ago2 in puncta. Note the absence of Dcp1a or Pat1 puncta in G0, and the rapid reassembly in R3. Also note prominent nuclear staining for Edc4 in G0. Figure S2. (A) Supplementary to Figure 4A Additional representative immunofluorescence images showing Fmrp (green) and Dcp1a (red) puncta in [file 13395_2021_270_MOESM1_ESM.zip › Roy et al rev2 Fig S5_ESM.pdf]

Figure S4

**A**

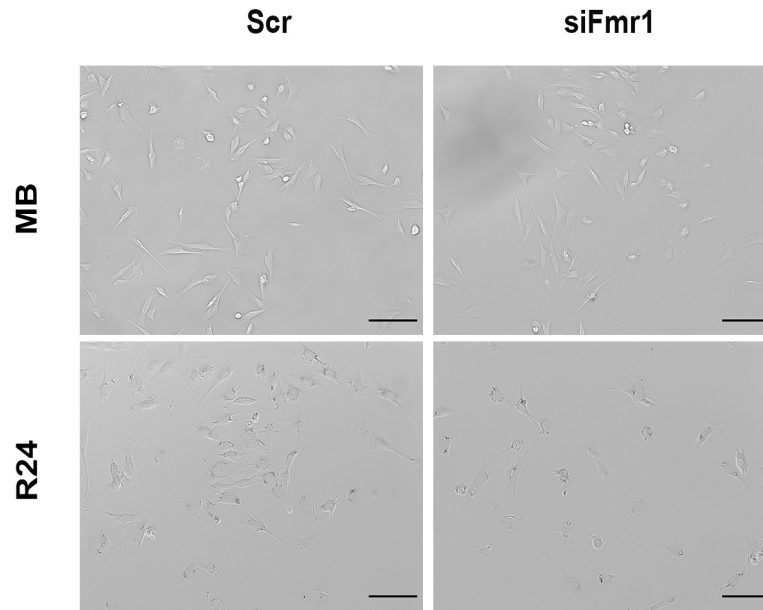

**B**

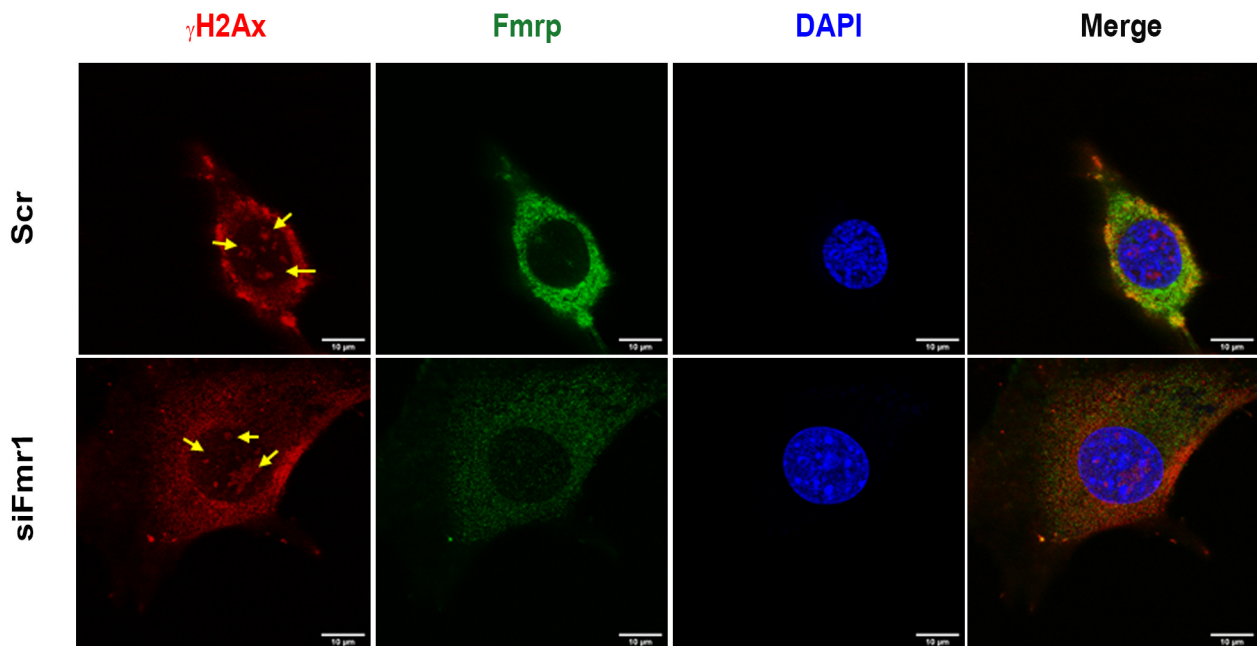

**C**

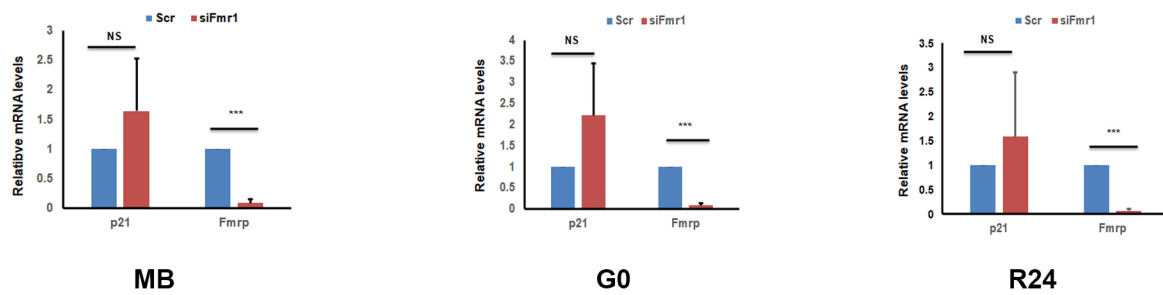

Supplement: Supplementary file 1 — Additional file 1: Table 1. Bio-informatic analysis of transcripts encoding mRNP components. To assess whether changes in expression of mRNP proteins resulted from changes in expression of their mRNAs, we used the recent RNA seq analysis derived from muscle satellite cells fixed by perfusion of adult mice (to prevent cell activation that results from disruption of the niche during isolation [42]. These fixed satellite cells are thought to more accurately represent the quiescent (G0 state) and have a transcriptome profile distinct from MuSC isolated without fixation, which are now understood to represent cells in an early activation state. Activated satellite cells (ASC) represent proliferating primary myoblasts 2.5 days post isolation from the animal. Transcripts encoding P body genes were selected from the RNAseq data and grouped according to their function as outlined [43, 44]. We calculated fold changes from FPKM values (Fragments Per Kilobase of transcript per Million mapped reads) RNA seq data comparing fixed (quiescent) satellite cells and activated satellite cells [44] and used a cut-off of 1.5 +/- (for up regulation and down regulation). False Discovery Rate approach: Two stage step-up method of Benjamini, Krieger and Yekutieli was used and 10% FDR was set up for generating p values for the analysis. Figure S1. Differential association of decay complex proteins in different cellular states. Immuno-staining of Dcp1a/Edc4/Pat1 (left) and Dcp1a/Ago2 (right) in muscle cells in culture: quiescent (G0), 3 hr reactivated (R3), proliferative (MB), and differentiated (MT). Blue arrows indicate co-localization of Dcp1a/Edc4/Pat1 in puncta. Red arrows indicate co-localization of Dcp1a/Ago2 in puncta. Note the absence of Dcp1a or Pat1 puncta in G0, and the rapid reassembly in R3. Also note prominent nuclear staining for Edc4 in G0. Figure S2. (A) Supplementary to Figure 4A Additional representative immunofluorescence images showing Fmrp (green) and Dcp1a (red) puncta in [file 13395_2021_270_MOESM1_ESM.zip › Roy et al rev2 Fig S4_ESM.pdf]

A

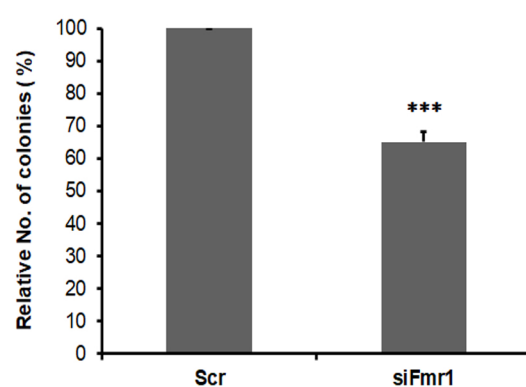

B

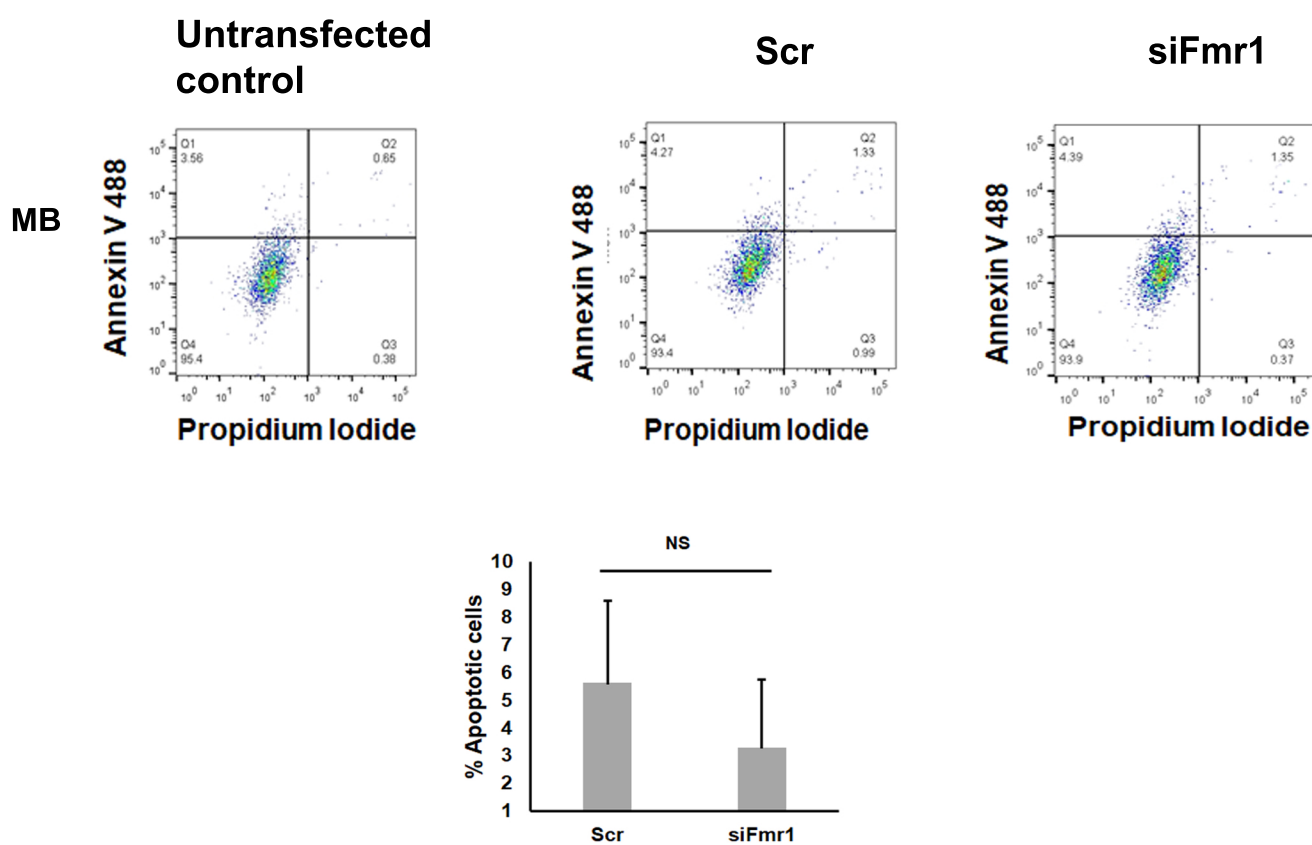

Supplement: Supplementary file 1 — Additional file 1: Table 1. Bio-informatic analysis of transcripts encoding mRNP components. To assess whether changes in expression of mRNP proteins resulted from changes in expression of their mRNAs, we used the recent RNA seq analysis derived from muscle satellite cells fixed by perfusion of adult mice (to prevent cell activation that results from disruption of the niche during isolation [42]. These fixed satellite cells are thought to more accurately represent the quiescent (G0 state) and have a transcriptome profile distinct from MuSC isolated without fixation, which are now understood to represent cells in an early activation state. Activated satellite cells (ASC) represent proliferating primary myoblasts 2.5 days post isolation from the animal. Transcripts encoding P body genes were selected from the RNAseq data and grouped according to their function as outlined [43, 44]. We calculated fold changes from FPKM values (Fragments Per Kilobase of transcript per Million mapped reads) RNA seq data comparing fixed (quiescent) satellite cells and activated satellite cells [44] and used a cut-off of 1.5 +/- (for up regulation and down regulation). False Discovery Rate approach: Two stage step-up method of Benjamini, Krieger and Yekutieli was used and 10% FDR was set up for generating p values for the analysis. Figure S1. Differential association of decay complex proteins in different cellular states. Immuno-staining of Dcp1a/Edc4/Pat1 (left) and Dcp1a/Ago2 (right) in muscle cells in culture: quiescent (G0), 3 hr reactivated (R3), proliferative (MB), and differentiated (MT). Blue arrows indicate co-localization of Dcp1a/Edc4/Pat1 in puncta. Red arrows indicate co-localization of Dcp1a/Ago2 in puncta. Note the absence of Dcp1a or Pat1 puncta in G0, and the rapid reassembly in R3. Also note prominent nuclear staining for Edc4 in G0. Figure S2. (A) Supplementary to Figure 4A Additional representative immunofluorescence images showing Fmrp (green) and Dcp1a (red) puncta in [file 13395_2021_270_MOESM1_ESM.zip › Roy et al rev2 Fig S3_ESM.pdf]

Figure S1

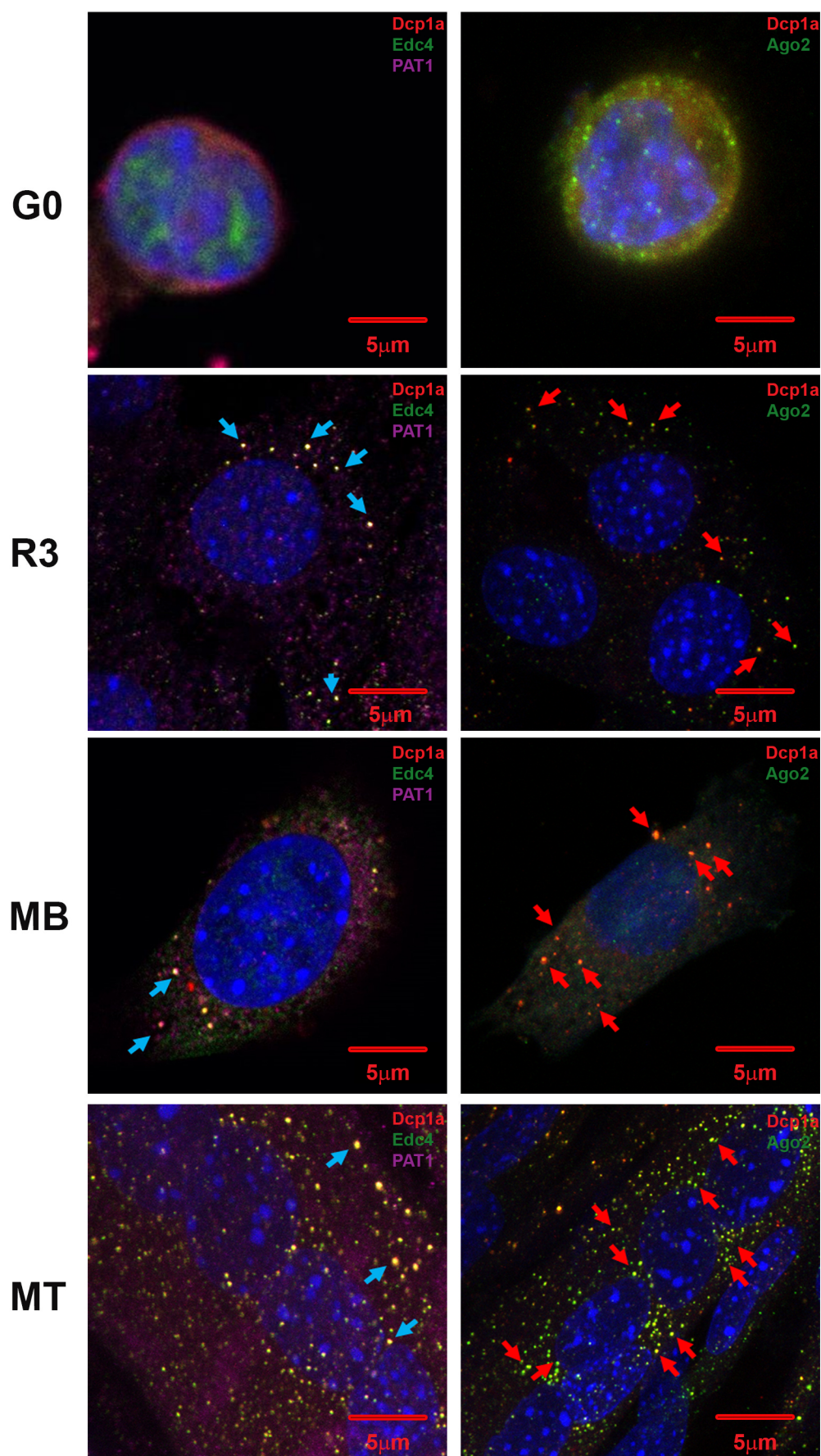

Supplement: Supplementary file 1 — Additional file 1: Table 1. Bio-informatic analysis of transcripts encoding mRNP components. To assess whether changes in expression of mRNP proteins resulted from changes in expression of their mRNAs, we used the recent RNA seq analysis derived from muscle satellite cells fixed by perfusion of adult mice (to prevent cell activation that results from disruption of the niche during isolation [42]. These fixed satellite cells are thought to more accurately represent the quiescent (G0 state) and have a transcriptome profile distinct from MuSC isolated without fixation, which are now understood to represent cells in an early activation state. Activated satellite cells (ASC) represent proliferating primary myoblasts 2.5 days post isolation from the animal. Transcripts encoding P body genes were selected from the RNAseq data and grouped according to their function as outlined [43, 44]. We calculated fold changes from FPKM values (Fragments Per Kilobase of transcript per Million mapped reads) RNA seq data comparing fixed (quiescent) satellite cells and activated satellite cells [44] and used a cut-off of 1.5 +/- (for up regulation and down regulation). False Discovery Rate approach: Two stage step-up method of Benjamini, Krieger and Yekutieli was used and 10% FDR was set up for generating p values for the analysis. Figure S1. Differential association of decay complex proteins in different cellular states. Immuno-staining of Dcp1a/Edc4/Pat1 (left) and Dcp1a/Ago2 (right) in muscle cells in culture: quiescent (G0), 3 hr reactivated (R3), proliferative (MB), and differentiated (MT). Blue arrows indicate co-localization of Dcp1a/Edc4/Pat1 in puncta. Red arrows indicate co-localization of Dcp1a/Ago2 in puncta. Note the absence of Dcp1a or Pat1 puncta in G0, and the rapid reassembly in R3. Also note prominent nuclear staining for Edc4 in G0. Figure S2. (A) Supplementary to Figure 4A Additional representative immunofluorescence images showing Fmrp (green) and Dcp1a (red) puncta in [file 13395_2021_270_MOESM1_ESM.zip › Roy et al rev2 Fig S1_ESM.pdf]
